# Findings From the World Mental Health Surveys of Civil Violence Exposure and Its Association With Subsequent Onset and Persistence of Mental Disorders

**DOI:** 10.1001/jamanetworkopen.2023.18919

**Published:** 2023-06-20

**Authors:** William G. Axinn, Ronny Bruffaerts, Timothy L. Kessler, Rochelle Frounfelker, Sergio Aguilar-Gaxiola, Jordi Alonso, Brendan Bunting, José Miguel Caldas-de-Almeida, Graça Cardoso, Stephanie Chardoul, Wai Tat Chiu, Alfredo Cía, Oye Gureje, Elie G. Karam, Viviane Kovess-Masfety, Maria V. Petukhova, Marina Piazza, José Posada-Villa, Nancy A. Sampson, Kate M. Scott, Juan Carlos Stagnaro, Dan J. Stein, Yolanda Torres, David R. Williams, Ronald C. Kessler

**Affiliations:** 1 Survey Research Center, University of Michigan, Ann Arbor; 2 Universitair Psychiatrisch Centrum–Katholieke Universiteit, Campus Gasthuisberg, Leuven, Belgium; 3 Department of Health Care Policy, Harvard Medical School, Boston, Massachusetts; 4 Department of Community and Population Health, Lehigh University, Bethlehem, Pennsylvania; 5 Center for Reducing Health Disparities, University of California, Davis Health System, Sacramento; 6 Health Services Research Group, Hospital del Mar Medical Research Institute, Barcelona, Spain; 7 Biomedical Research Networking Center in Epidemiology and Public Health, Barcelona, Spain; 8 Department of Medicine and Life Sciences, Universitat Pompeu Fabra, Barcelona, Spain; 9 School of Psychology, Ulster University, Londonderry, United Kingdom; 10 Lisbon Institute of Global Mental Health and Comprehensive Health Research Centre, NOVA Medical School, Universidade Nova de Lisboa, Lisbon, Portugal; 11 Anxiety Disorders Research Center, Buenos Aires, Argentina; 12 Department of Psychiatry, University College Hospital, Ibadan, Nigeria; 13 Department of Psychiatry and Clinical Psychology, Saint George Hospital University Medical Center, Beirut, Lebanon; 14 Faculty of Medicine, University of Balamand, Beirut, Lebanon; 15 Institute for Development, Research, Advocacy and Applied Care, Beirut, Lebanon; 16 Institut de Psychologie, Université Paris Cité, Paris, France; 17 Instituto Nacional de Salud, Universidad Cayetano Heredia, Lima, Peru; 18 Faculty of Social Sciences, Colegio Mayor de Cundinamarca University, Bogota, Colombia; 19 Department of Psychological Medicine, University of Otago, Dunedin, New Zealand; 20 Departamento de Psiquiatría y Salud Mental, Facultad de Medicina, Universidad de Buenos Aires, Buenos Aires, Argentina; 21 South African Medical Council Research Unit on Risk and Resilience in Mental Disorders, Department of Psychiatry and Mental Health, University of Cape Town and Groote Schuur Hospital, Cape Town, South Africa; 22 Center for Excellence on Research in Mental Health, CES University, Medellín, Colombia; 23 Department of Social and Behavioral Sciences, Harvard T.H. Chan School of Public Health, Boston, Massachusetts

## Abstract

**Importance:**

Understanding the association of civil violence with mental disorders is important for developing effective postconflict recovery policies.

**Objective:**

To estimate the association between exposure to civil violence and the subsequent onset and persistence of common mental disorders (in the *Diagnostic and Statistical Manual of Mental Disorders, Fourth Edition* [*DSM-IV*]) in representative surveys of civilians from countries that have experienced civil violence since World War II.

**Design, Setting, and Participants:**

This study used data from cross-sectional World Health Organization World Mental Health (WMH) surveys administered to households between February 5, 2001, and January 5, 2022, in 7 countries that experienced periods of civil violence after World War II (Argentina, Colombia, Lebanon, Nigeria, Northern Ireland, Peru, and South Africa). Data from respondents in other WMH surveys who immigrated from countries with civil violence in Africa and Latin America were also included. Representative samples comprised adults (aged ≥18 years) from eligible countries. Data analysis was performed from February 10 to 13, 2023.

**Exposures:**

Exposure was defined as a self-report of having been a civilian in a war zone or region of terror. Related stressors (being displaced, witnessing atrocities, or being a combatant) were also assessed. Exposures occurred a median of 21 (IQR, 12-30) years before the interview.

**Main Outcomes and Measures:**

The main outcome was the retrospectively reported lifetime prevalence and 12-month persistence (estimated by calculating 12-month prevalence among lifetime cases) of *DSM-IV* anxiety, mood, and externalizing (alcohol use, illicit drug use, or intermittent explosive) disorders.

**Results:**

This study included 18 212 respondents from 7 countries. Of these individuals, 2096 reported that they were exposed to civil violence (56.5% were men; median age, 40 [IQR, 30-52] years) and 16 116 were not exposed (45.2% were men; median age, 35 [IQR, 26-48] years). Respondents who reported being exposed to civil violence had a significantly elevated onset risk of anxiety (risk ratio [RR], 1.8 [95% CI, 1.5-2.1]), mood (RR, 1.5 [95% CI, 1.3-1.7]), and externalizing (RR, 1.6 [95% CI, 1.3-1.9]) disorders. Combatants additionally had a significantly elevated onset risk of anxiety disorders (RR, 2.0 [95% CI, 1.3-3.1]) and refugees had an increased onset risk of mood (RR, 1.5 [95% CI, 1.1-2.0]) and externalizing (RR, 1.6 [95% CI, 1.0-2.4]) disorders. Elevated disorder onset risks persisted for more than 2 decades if conflicts persisted but not after either termination of hostilities or emigration. Persistence (ie, 12-month prevalence among respondents with lifetime prevalence of the disorder), in comparison, was generally not associated with exposure.

**Conclusions:**

In this survey study of exposure to civil violence, exposure was associated with an elevated risk of mental disorders among civilians for many years after initial exposure. These findings suggest that policy makers should recognize these associations when projecting future mental disorder treatment needs in countries experiencing civil violence and among affected migrants.

## Key Points

**Question:**

How is exposure to civil violence (in a war zone or region of terror) associated with the onset or persistence of common mental disorders among civilians in countries that have experienced civil violence since World War II?

**Findings:**

In this survey study including 18 212 respondents in 7 countries, personal exposure to civil violence was associated with a significantly increased risk of onset of diverse mental disorders. These associations persisted for decades but not after termination of hostilities or emigration; associations with disorder persistence were generally nonsignificant.

**Meaning:**

These findings suggest that policy makers should recognize the associations between civil violence and mental health outcomes when projecting future treatment needs of civilians.

## Teaser

This survey study uses World Mental Health survey data from countries that have experienced civil violence since World War II to assess the association between such exposure and subsequent onset and persistence of mental disorders among civilians.

## Introduction

The war in Ukraine has brought renewed attention to the mental health of war-affected populations.^1,2^ But Ukraine is far from the only country experiencing war or civil violence. The World Bank estimates that more than 1 billion people worldwide currently live in regions affected by armed conflict, an increase of 200 million since 2012.^3^ The United Nations High Commissioner for Refugees estimates that more than 100 million civilians are now forcibly displaced from their homes due to war or civil violence.^4^ Understanding the association between these experiences and mental health outcomes is vital to designing and implementing policies and programs both during conflict and in postconflict settings and to improving estimates of the societal costs of these conflicts.

Information on the mental health consequences of war and civil violence comes largely,^5,6,7,8^ although not entirely,^9,10^ from studies of individuals from conflict-affected countries in the few years after the conflicts have ended. These studies document a high prevalence of mental disorders, particularly posttraumatic stress disorder and depression. But an accurate account of the mental health costs of these conflicts also needs to take into consideration long-term mental health trajectories. Although research on the latter topic is limited, studies of World War II veterans,^11,12^ Holocaust survivors,^13,14^ and children evacuated during wartime show that clinically significant psychological distress often persists for many years.^15,16^

Fewer studies have examined long-term mental health outcomes in representative samples of all individuals from countries experiencing war or civil violence.^17,18^ One exception was a study carried out in the World Health Organization World Mental Health (WMH) surveys of mental disorder prevalence many years later with civilians who lived in a war zone during World War II.^19^ Substantially elevated disorder prevalence was documented in that study.

The current study used data from the WMH surveys to extend the earlier analysis to consider civil conflicts since World War II. We focused on 3 issues: (1) associations of exposure to civil violence (defined as self-reports of being a civilian either in a war zone or region of terror) in countries that experienced periods of civil violence with subsequent onset of common mental disorders; (2) associations of exposure to civil violence with subsequent persistence of these disorders; and (3) variation in these associations by self-reported age of first exposure and time since exposure, whether the hostilities were still ongoing or had ended as of the time of interview, and whether the respondent emigrated to another country. Based on the aforementioned prior research, we anticipated that personally being exposed to civil violence would be associated with a significantly elevated risk of lifetime mental disorders that would decay over time but persist for many years.

## Methods

### Sample

This survey study used data from the WMH surveys administered to households between February 5, 2001, and January 5, 2022. Thus far, these surveys have been administered face to face in representative household samples of adults (aged ≥18 years) in 29 countries throughout the world (eTable 1 in Supplement 1). Informed consent was obtained in all surveys based on procedures approved by the institutional review boards of the organizations that implemented the surveys. Details about the WMH design and field procedures are described elsewhere.^20^ Of the 29 WMH countries, 7 have experienced periods of civil violence in the years since World War II: Argentina, Colombia, Lebanon, Nigeria, Northern Ireland, Peru, and South Africa. The WMH survey sample sizes varied between 781 and 4077 within a country (n = 15 525 total). In addition, meaningful numbers of WMH respondents in other surveys immigrated from countries that experienced civil violence in Latin America (n = 2601), including Bolivia, Brazil, Chile, El Salvador, Guatemala, Honduras, Mexico, Nicaragua, and Uruguay, and in Africa (n = 86), including Algeria, Angola, Congo, Guinea-Bissau, and Mozambique. The current study focused on these 18 212 WMH respondents.

Informed consent was obtained before beginning interviews. Procedures for obtaining informed consent were different across countries but were always approved by the institutional review boards of the collaborating organizations in each country. Only deidentified data were deposited in the centralized WMH server. Analyses were carried out on that server by trained and approved WMH analysts. This report followed the American Association for Public Opinion Research (AAPOR) reporting guideline for survey studies.

### Measures

#### Exposure to Civil Violence

Respondents were asked a series of yes-or-no questions about lifetime exposure to experiences that were conceptualized in the interviews as traumatic according to the criteria of the *Diagnostic and Statistical Manual of Mental Disorders, Fourth Edition* (*DSM-IV*) or *International Statistical Classification of Diseases, Tenth Revision* systems, although not all of these experiences fulfill the *Diagnostic and Statistical Manual of Mental Disorders, Fifth Edition* (*DSM-5*) requirement of “actual or threatened death, serious injury, or sexual violence.” Two of these questions were as follows: “Were you ever an unarmed civilian in a place where there was a war, revolution, military coup or invasion?” and “Did you ever live as a civilian in a place where there was ongoing terror of civilians for political, ethnic, religious or other reasons?” Respondents who answered positively were asked their age at first exposure. Missing values were coded “no.” If the reported year of disorder onset corresponded to a year in which a civil conflict was known to have occurred in the respondent’s country of residence (the eAppendix in Supplement 1 provides an overview of these conflicts and their years of occurrence), the respondent was included in the analysis. In the small number of cases in which the reported year of disorder onset was either missing or outside these years (n = 155), the respondent was excluded from the analysis. We also asked all respondents about exposure and age of first exposure to 3 related stressors: being a combatant (“Did you ever participate in combat, either as a member of a military, or as a member of an organized non-military group?”), becoming a refugee (“Were you ever a refugee—that is, did you ever flee from your home to a foreign country or place to escape danger or persecution?”), and witnessing atrocities (“Did you ever see atrocities or carnage such as mutilated bodies or mass killings?”).

#### Mental Disorders

The WMH surveys used the WHO Composite International Diagnostic Interview (CIDI), version 3.0,^21^ to assess the lifetime and 12-month presence of *DSM-IV* anxiety disorders (generalized anxiety disorder, panic disorder with or without agoraphobia, posttraumatic stress disorder, specific phobia, or social phobia), mood disorders (bipolar spectrum disorder or major depressive disorder), and externalizing disorders (alcohol use disorder, illicit drug use disorder, or intermittent explosive disorder). Item-missing symptom reports were coded as if the symptoms were not present. Good concordance was observed between these CIDI diagnoses and independent blinded clinical diagnoses.^22^ Lifetime disorder age of onset was determined by retrospective recall using special probing techniques designed to optimize the accuracy of dating.^23^ Respondent reports of uncertainty in recalling age of onset were probed by asking a series of yes-or-no questions about approximate age ranges (eg, “Was it before you were a teenager?” If not, then the individual was asked, “Was it before you were 20 years old?”) along with a question about the earliest age the respondent could “clearly remember” having the disorder.

### Statistical Analysis

Associations of personal exposure to civil violence with the subsequent first onset of mental disorders were estimated with discrete-time survival analysis using a log link function.^24,25^ Given that retrospective dates of the first exposure to civil violence and the onset of each disorder were both obtained by age in years, a discrete-time survival analysis with person-year as the unit of analysis was chosen to analyze the data rather than a continuous-time approach. Survival coefficients and their SEs were exponentiated to create risk ratios (RRs) and 95% CIs. Respondents with onset of a given disorder prior to the beginning of the period of civil violence in the country (dates provided in Supplement 1) were excluded from analysis of that specific disorder, as our interest was in the association between personal exposure to the violence and subsequent first onset of the disorder among respondents with no prior history of the disorder. The same respondents were included for other disorders unless the same issue of prior onset occurred. Associations of exposure with disorder persistence were estimated at the person level, again using a log link function, with the outcome defined as the 12-month prevalence among lifetime cases controlling for disorder age of onset and time since onset. Interaction analyses examined variation in RRs of onset and persistence as a function of age at exposure, number of years since exposure, whether hostilities were still ongoing or had ended, and whether the respondent had emigrated to another country. Statistical significance was evaluated consistently with 2-sided Wald χ^2^ tests at the .05 level. Data analysis was performed February 10 to 13, 2023.

## Results

### Sample Distributions

Of the 18 212 survey respondents who lived in countries during the years when civil violence occurred, 2096 reported being personally exposed to civil violence (56.5% were men and 43.5% were women; median age, 40 [IQR, 30-52] years) and 16 116 were not exposed (45.2% were men and 54.8% were women; median age, 35 [IQR, 26-48] years) (Table 1). The median age at first exposure was 18 (IQR, 11-27) years, and the median time between first exposure and age at interview was 21 (IQR, 12-30) years. Of the respondents who were exposed, 28.2% also experienced 1 or more of the related stressors (17.4% became refugees, 10.4% witnessed atrocities, and 6.0% became combatants).

**Table 1.** Sociodemographic and Related Stressor Characteristics of Respondents Exposed to Civil Violence

Sociodemographic and stressor	Value
Total No. of respondents	2096
Sex, % (SE)^a^	
Men	56.5 (1.1)
Women	43.5 (1.1)
Age, median (IQR), y	
At interview^a^	40 (30-52)
At first exposure	18 (11-27)
Time between first exposure and interview, median (IQR), y	21 (12-30)
Related stressor, % (SE)	
Refugee	17.4 (0.8)
Saw atrocities	10.4 (0.7)
Combatant	6.0 (0.5)
Any of the 3	28.2 (1.0)

^a^ Comparable distributions (SEs) among the 16 116 not exposed were 45.2% (0.4) for men and 54.8% (0.4) for women, with a median age at interview of 35 (IQR, 26-48) years.

Although 10.6% of eligible WMH respondents (ie, living in countries where civil violence occurred) across surveys reported personal exposure to civil violence, this exposure ranged between a high of 59.9% in Lebanon and a low of 1.8% among respondents who immigrated from other Latin American countries than those in the WMH series (Table 2). That only a minority of the people living in these countries reported personal exposure is consistent with the prior WMH study of people living in countries directly involved in fighting during World War II.^19^

**Table 2.** World Mental Health Survey Samples and Proportions Directly Exposed to Civil Violence

Country	Proportion exposed, % (SE)^a^	No. of respondents^b^	
		Exposed (n = 2096)	Not exposed (n = 16 116)
Surveyed			
Argentina	3.4 (0.5)	78	1358
Colombia	9.0 (0.5)	462	3433
Lebanon	59.9 (1.8)	516	265
Nigeria	7.9 (0.6)	154	1664
Northern Ireland	21.0 (1.0)	387	1344
Peru	6.6 (0.6)	128	1659
South Africa	8.5 (0.4)	286	3791
Other country of origin			
Other country in Latin America^c^	1.8 (0.3)	49	2552
Other country in Africa^d^	37.7 (5.3)	36	50

Abbreviation: WMH, World Mental Health.

^a^ Proportion of respondents in the survey carried out in the country in the row heading who reported that they had been a civilian either in a war zone or a region of terror. The percentage is based on weighted numbers that adjusted for differential probabilities of selection across respondents due to selecting only 1 respondent per household no matter how many eligible people lived in the household and that calibrated to population sociodemographic and geographic distributions. The total proportion (SE) exposed was 10.6% (0.2).

^b^ Exposed includes respondents who reported personally being exposed to civil violence, whereas not exposed includes respondents who reported not being personally exposed to civil violence but who lived in the same country during the same time period (±5 years of the time others in the same country were exposed) as those exposed.

^c^ Respondents who were born in other countries in Latin America that experienced civil violence and lived in those countries at the time these conflicts were taking place, but subsequently emigrated to a country where a WMH survey was carried out. The countries included Bolivia, Brazil, Chile, El Salvador, Guatemala, Honduras, Mexico, Nicaragua, and Uruguay.

^d^ Respondents who were born in other countries in Africa that experienced civil violence and lived in those countries at the time these conflicts were taking place, but subsequently emigrated to a country where a WMH survey was carried out. The countries included Algeria, Angola, Congo, Guinea-Bissau, and Mozambique.

### Associations of Exposure With Subsequent First Lifetime Onset of Mental Disorders

Gross RRs (ie, controlling only for person-year, country, and sex) of exposure to civil violence with subsequent first onset of *DSM-IV*/CIDI disorders were consistently significant and elevated, ranging from 1.8 (95% CI, 1.3-2.4) to 3.4 (95% CI, 2.6-4.5) (Table 3). Net RRs (ie, additionally controlling for prior lifetime onset of other disorders) were also consistently elevated across disorders and for the most part were statistically significant, ranging from 1.2 (95% CI, 0.8-1.9) to 2.4 (95% CI, 1.8-3.3).

**Table 3.** Associations of Exposure to Civil Violence With Subsequent First Onset of *Diagnostic and Statistical Manual of Mental Disorders, Fourth Edition*/CIDI Disorders^a^

Disorder	Lifetime disorder prevalence among respondents, % (SE)		Association of exposure with subsequent disorder onset, RR (95% CI)	
	Exposed^b^	Not exposed^c^	Gross^d^	Net^e^
Anxiety				
Generalized anxiety disorder	4.3 (0.4)	2.5 (0.1)	2.3 (1.8-3.1)^f^	1.7 (1.3-2.3)^f^
Panic and/or agoraphobia	2.8 (0.4)	4.1 (0.2)	1.8 (1.3-2.4)^f^	1.3 (0.9-1.8)
Posttraumatic stress disorder	5.0 (0.5)	2.1 (0.1)	3.4 (2.6-4.5)^f^	2.4 (1.8-3.3)^f^
Specific phobia	3.6 (0.4)	6.4 (0.2)	2.3 (1.5-3.5)^f^	2.1 (1.4-3.3)^f^
Social phobia	2.6 (0.4)	3.0 (0.1)	2.5 (1.8-3.6)^f^	1.8 (1.3-2.7)^f^
Any	12.4 (0.8)	14.0 (0.3)	2.5 (2.2-2.9)^f^	1.9 (1.6-2.2)^f^
Mood				
Bipolar spectrum disorder	1.9 (0.3)	1.4 (0.1)	2.0 (1.4-2.9)^f^	1.2 (0.8-1.9)
Major depressive disorder	12.5 (0.7)	8.7 (0.2)	2.0 (1.7-2.3)^f^	1.6 (1.4-1.9)^f^
Any	14.4 (0.8)	10.1 (0.2)	2.0 (1.7-2.3)^f^	1.5 (1.3-1.8)^f^
Externalizing				
Alcohol use disorder	10.4 (0.7)	8.0 (0.2)	1.8 (1.4-2.3)^f^	1.5 (1.2-1.9)^f^
Illicit substance use disorder	3.3 (0.4)	2.2 (0.1)	2.7 (1.9-3.7)^f^	2.0 (1.4-2.9)^f^
Intermittent explosive disorder	2.7 (0.4)	2.0 (0.1)	2.4 (1.8-3.4)^f^	1.9 (1.4-2.8)^f^
Any	15.9 (0.8)	10.4 (0.2)	2.1 (1.8-2.5)^f^	1.7 (1.4-2.0)^f^
Any disorder	28.0 (1.1)	26.1 (0.3)	2.2 (2.0-2.4)^f^	1.7 (1.6-1.9)^f^

Abbreviations: CIDI, Composite International Diagnostic Interview; RR, risk ratio.

^a^ Based on discrete-time survival models with person-year as the unit of analysis and a log link function transformed to generate RRs.

^b^ Conditional lifetime prevalence of the disorder subsequent to age of first exposure to civil violence in the subset of respondents who did not already have a lifetime history of the disorder prior to age of first exposure.

^c^ Unconditional prevalence of the disorder among respondents not exposed to civil violence.

^d^ Controlling for person-year, country, and respondent sex. Note that RR can be elevated even when prevalence is not higher among the exposed than the not exposed because prevalence among the exposed is conditional and among the not exposed is unconditional. This is adjusted for in the survival analyses that estimate RR.

^e^ Controlling for person-year, country, respondent sex, and temporally prior lifetime occurrence of all other disorders.

^f^ Significant at the .05 level, 2-sided test.

Pooled analyses also yielded consistently elevated, and, for the most part, statistically significant RRs of the related stressors with subsequent first onsets were observed in univariable models (ie, models that considered only 1 stressor at a time) for anxiety disorders, ranging from 1.8 (95% CI, 1.3-2.4) to 2.3 (95% CI, 1.5-3.4) for being a refugee, from 1.1 (95% CI, 0.6-1.9) to 3.2 (95% CI, 2.1-4.8) for being a combatant, and from 1.5 (95% CI, 1.0-2.1) to 2.1 (95% CI, 1.5-2.9) for seeing atrocities (Table 4). The RRs remained significant in multivariable models (ie, models that considered all stressors at once) for exposure to civil violence being associated with all 3 types of disorders (anxiety, 1.8 [95% CI, 1.5-2.1]; mood, 1.5 [95% CI, 1.3-1.7], externalizing, 1.6 [95% CI, 1.3-1.9]), being a combatant in association with anxiety disorders (2.0 [95% CI, 1.3-3.1]), and becoming a refugee in association with mood (1.5 [95% CI, 1.1-2.0]) and externalizing (1.6 [95% CI, 1.0-2.4]) disorders. These results were broadly similar in each of the 4 countries where the sample was large enough for within-country analysis (eTable 2 in Supplement 1).

**Table 4.** Risk Ratios of Subsequent Anxiety, Mood, and Externalizing Disorder Onset Associated With Exposure to Civil Violence and Related Stressors^a^

Stressor	Risk ratio (95% CI)		
	Any anxiety disorder	Any mood disorder	Any externalizing disorder
Univariable association^b^			
Exposed to civil violence	1.9 (1.6-2.2)^c^	1.5 (1.3-1.8)^c^	1.7 (1.4-2.0)^c^
Related stressors among those exposed to civil violence			
Became a refugee^b^	1.8 (1.3-2.4)^c^	2.0 (1.9-2.6)^c^	2.3 (1.5-3.4)^c^
Saw atrocities^b^	2.1 (1.5-2.9)^c^	1.5 (1.0-2.1)^c^	1.5 (1.0-2.3)^c^
Became a combatant^b^	3.2 (2.1-4.8)^c^	1.1 (0.6-1.9)	1.8 (0.9-3.5)
Any of the 3^b^	2.2 (1.8-2.9)^c^	1.6 (1.3-2.1)^c^	2.0 (1.5-2.9)^c^
Multivariable association^d^			
Exposed to civil violence	1.8 (1.5-2.1)^c^	1.5 (1.3-1.7)^c^	1.6 (1.3-1.9)^c^
Related stressors among those exposed to civil violence			
Became a refugee	1.1 (0.8-1.5)	1.5 (1.1-2.0)^c^	1.6 (1.0-2.4)^c^
Saw atrocities	1.1 (0.8-1.6)	1.1 (0.8-1.6)	1.0 (0.6-1.6)
Became a combatant	2.0 (1.3-3.1)^c^	0.8 (0.4-1.4)	1.2 (0.6-2.5)

^a^ Based on the net discrete-time survival models in Table 3 stacked across disorders.

^b^ Only 1 of the 4 stressors (ie, either exposure to civil violence, becoming a refugee, seeing atrocities, or becoming a combatant) was included in the model.

^c^ Significant at the .05 level, 2-sided test.

^d^ All stressors were included in the model.

Interaction analyses examined whether associations of exposure with subsequent first onset varied depending on whether the respondent was a child (aged 0-12 years), adolescent (aged 13-21 years), or adult (aged ≥22 years) at the time of exposure (Table 5). These interactions were for the most part nonsignificant (χ^2^_2_ = 0.4-4.8, *P* = .81-.09). The exception was that the RR for civil violence with onset of mood disorders was significant only when exposure began in childhood or adolescence (from 1.7 [95% CI, 1.3-2.1] to 1.7 [95% CI, 1.4-2.1]; χ^2^_1_ = 21.3-25.1, *P* < .001), not in adulthood (RR, 1.0 [95% CI, 0.8-1.2]; χ^2^_1_ = 0.0, *P* = .83). Within-country samples were too small to examine these age differences.

**Table 5.** Subgroup Variation in Significant Multivariable Associations of Exposure to Civil Violence and Related Stressors With Subsequent First Onset of Anxiety, Mood, and Externalizing Disorders as a Function of Age at Exposure to the Stressor^a^

Subgroup^b^	Risk ratio (95% CI)		
	Any anxiety disorder^c^	Any mood disorder	Any externalizing disorder
Age at exposure to civil violence, y			
0-12	2.0 (1.6-2.5)^d^	1.7 (1.3-2.1)^d^	1.5 (1.2-1.9)^d^
13-21	1.8 (1.5-2.2)^d^	1.7 (1.4-2.1)^d^	1.7 (1.3-2.2)^d^
≥22	1.4 (1.1-1.8)^d^	1.0 (0.8-1.2)	1.6 (1.1-2.4)^d^
χ^2^_3_	65.6^d^	39.7^d^	27.6^d^
χ^2^_2_	4.8	16.9^d^	0.7
Age at first becoming a refugee, y^e^			
0-12	NA	2.0 (1.3-3.2)^d^	2.3 (1.2-4.4)^d^
13-21	NA	1.2 (0.7-1.9)	1.3 (0.7-2.5)
≥22	NA	1.4 (0.9-2.2)	1.3 (0.6-2.8)
χ^2^_3_	NA	11.5^d^	7.9^d^
χ^2^_2_	NA	3.3	2.0

Abbreviation: NA, not applicable.

^a^ Based on the multivariable discrete-time survival models in Table 4 but with a decomposition of the significant stressor measures by age of first occurrence. These dummy variables were “turned on” at age of first occurrence and were time invariant across subsequent person-years.

^b^ χ^2^ Tests of the significance of the associations between the stressor measures and the outcome. The χ^2^_3_ tests with 3 degrees of freedom evaluated the significance of the set of 3 dummy variables, whereas the χ^2^_2_ tests with 2 degrees of freedom evaluated the significance of the differences across these 3 variables. The existence of significant variation in associations as a function of age at exposure would be expected to have a significant result in the χ^2^ test with 2 degrees of freedom.

^c^ Being a combatant was also significant in Table 4 for any anxiety disorder and was consequently included here as well. The risk ratio of being a combatant with anxiety disorder was 1.8 (95% CI, 0.2-3.5) for children aged 0 to 12 years, 2.0 (95% CI, 1.3-3.2) for adolescents aged 13 to 21 years, and 2.7 (95% CI, 1.1-6.7) for adults aged 22 years or older, with χ^2^_3_ = 13.3 and χ^2^_3_ = 0.4.

^d^ Significant at the .05 level, 2-sided test.

^e^ As shown in Table 4, being a refugee was not associated with anxiety disorders and consequently was not included here in the model for anxiety disorders.

We also examined whether associations of exposure to civil violence with subsequent first disorder onset varied with number of years since first exposure (divided into 5-year intervals). This difference was significant for mood (χ^2^_6_ = 79.1, *P* < .001) and externalizing (χ^2^_6_ = 48.8, *P* < .001) disorders but not for anxiety disorders (χ^2^_6_ = 9.8, *P* = .13) (eTable 3 in Supplement 1), as RR decreased with increasing time for both mood (0-5 years: 6.4 [95% CI, 3.6-11.5]; and ≥31 years: 0.9 [95% CI, 0.7-1.1]) and externalizing (0-5 years: 3.7 [95% CI, 1.3-10.5]; and ≥31 years: 0.9 [95% CI, 0.7-1.2]) disorders but remained consistently significant up through years 21 to 25 for both classes of disorders (mood: χ^2^_1_ = 8.1-43.3, *P* = .004-<.001; and externalizing: χ^2^_1_ = 6.3-57.4, *P* = .01-<.001). The samples were too small for similar analyses of related stressors or for within-country analyses of time since civil violence exposure.

Finally, we examined whether the significant associations of exposure to civil violence and the related stressors with subsequent disorder onset persisted even in the years after hostilities ended or if respondents emigrated to another country. For the most part, they did not (eTable 4 in Supplement 1). The exception was the association between becoming a refugee and subsequent onset of a mood disorder, which was elevated not only during the years when hostilities were ongoing (RR, 1.4 [95% CI, 1.0-1.9]; χ^2^_1_ = 4.2, *P* = .04) but also after the termination of hostilities (RR, 2.5 [95% CI, 1.3-4.5]; χ^2^_1_ = 8.6, *P* = .003). In all other cases, the significant associations documented here were restricted to the years when hostilities were ongoing and the respondent remained in the country of exposure (RR, 1.6 [95% CI, 1.1-2.4] to 2.2 [95% CI, 1.4-3.5]; χ^2^_1_ = 5.3-67.9, *P* = .02-<.001). The samples in individual countries were too sparse to allow replication of these specifications within countries.

### Associations of Exposure With Persistence of Lifetime Mental Disorders

After excluding cases with first lifetime onsets in the 2 years before interview, the 12-month persistence of disorders among lifetime cases was not associated with preonset history of exposure to civil violence. This was true of both gross (RR, 0.8 [95% CI, 0.5-1.2] to 1.2 [95% CI, 0.7-2.1]; χ^2^_1_ = 0.1-1.2, *P* = .77-.27) and net (RR, 0.8 [95% CI, 0.5-1.1] to 1.1 [95% CI, 0.6-1.9]; χ^2^_1_ = 0.0-1.7, *P* = .82-.19) associations (eTable 5 in Supplement 1). The same was largely true for pooled analyses of associations of related stressors with persistence (gross RR, 0.5 [95% CI, 0.2-1.3] to 1.1 [95% CI, 0.8-1.5]; χ^2^_1_ = 0.1-2.1, *P* = .75-.15; and net RR, 0.6 [95% CI, 0.2-1.4] to 1.3 [95% CI, 0.9-1.7]; χ^2^_1_ = 0.0-2.8, *P* = .91-.09; eTable 6 in Supplement 1). The one exception was a marginally significant negative univariable association between becoming a refugee and persistence of externalizing disorders (RR, 0.6 [95% CI, 0.3-1.0]; χ^2^_1_ = 3.9, *P* = .049).

We also examined whether the associations of exposure to civil violence with disorder persistence varied depending on respondent age at exposure. There was no evidence of such an association (χ^2^_2_ = 1.3-1.9, *P* = .53-.38) (eTable 7 in Supplement 1). In addition, we did not observe significant variation in the persistence of either mood (χ^2^_6_ = 4.5, *P* = .61) or externalizing (χ^2^_6_ = 8.8, *P* = .19) disorders depending on the number of years since first exposure. However, number of years since first exposure was associated with the persistence of anxiety disorders (χ^2^_6_ = 18.4, *P* = .005) due to a significantly elevated association of exposure in the 0 to 5 years before interview with 12-month persistence (RR, 2.1 [95% CI, 1.5-3.0]; χ^2^_1_ = 16.3, *P* < .001) (eTable 8 in Supplement 1). The samples were too small to investigate these associations within countries or to investigate similar associations involving related stressors (ie, refugee, atrocities, combatant).

## Discussion

In this study, we observed that being personally exposed to civil violence was associated with an elevated risk of onset of anxiety, mood, and externalizing disorders. Our findings also suggest that being a combatant was associated with an elevated risk of onset of anxiety disorders and that being a refugee was associated with an elevated risk of onset of mood and externalizing disorders. These elevated risks persisted for more than 2 decades after initial exposure if conflicts persisted but not after either termination of hostilities or emigration. Among lifetime cases, in comparison, disorder persistence was largely unrelated to prior exposure to civil violence.

As noted in the introduction, the global population living in regions exposed to civil violence is large and growing, with the World Bank estimating that 1.1 billion people (14% of the world’s population) lived in “fragile and conflict exposed situations”^3^ in 2020, compared with 612 million people (10% of the world’s population) in 2000. A previous study suggested that the absolute and relative growth in these populations resulted from high birthrates and young ages (due partly to early mortality) in conflict regions.^26^ Because of these forces, 40% of people living in areas exposed to civil conflict today are aged younger than 14 years compared with only 16% of people living in high-income countries.^27^ This observation makes our finding of an inverse association between age at first exposure and risk of onset of mental disorders all the more important. The conjunction of the relatively young age at time of exposure in the WMH data (median, 18 [IQR, 11-27] years) and the persistence of elevated risk for many years emphasize this durable association with age. Country-specific studies demonstrate similar long-lasting consequences of childhood exposure to civil violence. These results are consistent with other evidence of lasting consequences in additional overwhelmingly youthful countries that are experiencing civil conflicts.^28^

Related to these age-of-onset patterns, it is noteworthy that prior WMH studies found that early onset of mental disorders is associated with significant reductions in both education^29^ and earnings throughout the life course.^30^ This finding is important not only for individuals but also for postconflict societies, as civil conflicts are overwhelmingly concentrated in low and low- to middle-income countries where the pace of economic development not only remains tenuous but, in fact, has decreased over time.^27^ This observation suggests that new generations of young adults with a history of exposure to civil violence in already poor and unstable regions may become less economically productive, potentially contributing to a cycle of economic loss and civil conflict. It is consequently important to identify and address mental disorders both within these countries and among emigrants from these countries to support positive future economic, social, and political growth. Existing data document outcomes of scalable interventions to reduce trauma-induced mental disorders among children, adolescents, and adults.^31,32^ The results reported here argue indirectly that increasing efforts to screen for, and treat, these disorders in populations exposed to civil conflict may produce outsized benefits, with a special focus on individuals exposed to civil violence during their youth.

### Limitations

Seven important limitations of this study need to be highlighted. First, the sample was limited to people living in households at the time of interview. This means that some of the more than 100 million people estimated by the World Bank to be forcibly displaced at any time to escape violence, most notably those in refugee camps, were not represented in the sample,^33^ probably leading to an underestimation of association. Second, another source of underestimation of associations came from the fact that we compared respondents in the affected countries who reported that they were personally exposed with those in the same countries who were not personally exposed to civil violence. But even the people not directly exposed were nonetheless living under the threat of civil violence and were consequently likely to have a higher prevalence of mental disorders than individuals living in countries that were not at war. Third, although we observed broad consistency in results across the 4 WMH countries with samples large enough for within-country analysis (Colombia, Lebanon, Northern Ireland, and South Africa), the situations might have been different in conflict situations not included in the sample. Fourth, some of the WMH surveys were carried out more than 2 decades ago. The results might have been different if we had focused on more recent conflicts. Fifth, RR estimates might have been biased due to recall error or sample selection bias. Sixth, even if RR estimates were not biased, causal inferences cannot be made, given the observational study design. Seventh, given that civil violence was only one of many questions addressed in the WMH surveys, only a handful of questions were asked about this type of stressor. Much more extensive question series are used in studies that focus explicitly on refugees or other survivors of civil violence.^34,35^ A much clearer portrait of the consequences of exposure to civil violence would presumably be obtained with these more detailed measures. In addition, effect size estimates would presumably be larger with more detailed measures than with the coarse measure used here.

## Conclusions

In this survey study of exposure to civil violence, exposure was associated with an elevated risk of mental disorders for many years after initial exposure. To our knowledge, this study is the first to evaluate long-term lifetime risk of a range of mental disorders in cross-national general population household samples of individuals who were civilians in countries that experienced civil violence in the years since the end of World War II. Other studies of the association between exposure to war and mental disorders focused largely on the mental disorders of currently displaced people^36^ or survivors of recent conflicts.^37^ The few studies of long-term outcomes of war focused almost exclusively on current mental disorders.^38,39^ Our finding of elevated disorder onset risk is not surprising, but it is useful to know for service planning purposes that this risk was especially high among people first exposed during their youth, that this onset risk continued for many years among people living in countries that continued to have civil violence, that onset risk declined after the termination of hostilities and after emigration, and that disorder persistence was largely not associated with these factors.

## Notes

Supplement 1. **eAppendix.** Periods of Civil Conflicts in the Participating Countries **eTable 1.** World Mental Health (WMH) Sample Characteristics by World Bank Income Categories **eTable 2.** Significant Multivariable Associations of Exposure to Civil Violence and Related Stressors With Subsequent First Onset Anxiety, Mood, and Externalizing Disorders in World Mental Health (WMH) Surveys With Sufficiently Large Samples for Country-Specific Analysis **eTable 3.** Variation in Significant Multivariable Associations of Exposure to Civil Violence With Subsequent First Onset Anxiety, Mood, and Externalizing Disorder Onset as a Function of Number of Years Since First Exposure **eTable 4.** Variation in Significant Multivariable Associations of Exposure to Civil Violence and Related Stressors With Relative Risk of Subsequent Anxiety, Mood, and Externalizing Disorder Onset as a Function of Whether the Respondent Was Still Living in the Country and Hostilities Were Ongoing or Ended or Whether the Respondent Emigrated to Another Country **eTable 5.** Twelve-Month Persistence of *DSM-IV*/CIDI Disorders Among Lifetime Cases With Outsets at Least 2 Years Before Age at Interview and Relative Risk of 12-Month Disorder Persistence Associated With Being a Civilian in a War Zone **eTable 6.** Relative Risk of 12-Month *DSM-IV*/CIDI Anxiety, Mood, and Externalizing Disorder Persistence Among Lifetime Cases With Outsets at Least 2 Years Before Age at Interview Associated With Being a Civilian in a War Zone and Related Stressors **eTable 7.** Variation in Associations of Exposure to Civil Violence With Persistence of Subsequent Anxiety and Mood Disorders as a Function of Whether the Respondent Was Still Living in the Country and Hostilities Were Ongoing or Ended or Whether the Respondent Emigrated to Another Country **eTable 8.** Variation in Association of Exposure to Civil Violence With 12-Month Persistence of Subsequent Anxiety, Mood, and Externalizing Disorders as a Function of Number of Years Since First Exposure
eReferences

Supplement 2.
Nonauthor Collaborators

Supplement 3.
Data Sharing Statement
