# Supplementary material for: Findings From the World Mental Health Surveys of Civil Violence Exposure and Its Association With Subsequent Onset and Persistence of Mental Disorders
Source: JAMA Netw Open. 2023 Jun 20;6(6):e2318919. doi: 10.1001/jamanetworkopen.2023.18919 (PMC10282884; doi:10.1001/jamanetworkopen.2023.18919)
Supplement: Supplement 1. — eAppendix. Periods of Civil Conflicts in the Participating Countries eTable 1. World Mental Health (WMH) Sample Characteristics by World Bank Income Categories eTable 2. Significant Multivariable Associations of Exposure to Civil Violence and Related Stressors With Subsequent First Onset Anxiety, Mood, and Externalizing Disorders in World Mental Health (WMH) Surveys With Sufficiently Large Samples for Country-Specific Analysis eTable 3. Variation in Significant Multivariable Associations of Exposure to Civil Violence With Subsequent First Onset Anxiety, Mood, and Externalizing Disorder Onset as a Function of Number of Years Since First Exposure eTable 4. Variation in Significant Multivariable Associations of Exposure to Civil Violence and Related Stressors With Relative Risk of Subsequent Anxiety, Mood, and Externalizing Disorder Onset as a Function of Whether the Respondent Was Still Living in the Country and Hostilities Were Ongoing or Ended or Whether the Respondent Emigrated to Another Country eTable 5. Twelve-Month Persistence of DSM-IV/CIDI Disorders Among Lifetime Cases With Outsets at Least 2 Years Before Age at Interview and Relative Risk of 12-Month Disorder Persistence Associated With Being a Civilian in a War Zone eTable 6. Relative Risk of 12-Month DSM-IV/CIDI Anxiety, Mood, and Externalizing Disorder Persistence Among Lifetime Cases With Outsets at Least 2 Years Before Age at Interview Associated With Being a Civilian in a War Zone and Related Stressors eTable 7. Variation in Associations of Exposure to Civil Violence With Persistence of Subsequent Anxiety and Mood Disorders as a Function of Whether the Respondent Was Still Living in the Country and Hostilities Were Ongoing or Ended or Whether the Respondent Emigrated to Another Country eTable 8. Variation in Association of Exposure to Civil Violence With 12-Month Persistence of Subsequent Anxiety, Mood, and Externalizing Disorders as a Function of Number of Years Since First Exposure eReferences [file jamanetwopen-e2318919-s001.pdf]

Supplementary Online Content

Axinn WG, Bruffaerts R, Kessler TL, et al; WHO World Mental Health Survey Collaborators. Findings from the World Mental Health surveys of civil violence exposure and its association with subsequent onset and persistence of mental disorders. *JAMA Netw Open*. 2023;6(6):e2318919. doi:10.1001/jamanetworkopen.2023.18919

eAppendix. Periods of Civil Conflicts in the Participating Countries

eTable 1. World Mental Health (WMH) Sample Characteristics by World Bank Income Categories

eTable 2. Significant Multivariable Associations of Exposure to Civil Violence and Related Stressors With Subsequent First Onset Anxiety, Mood, and Externalizing Disorders in World Mental Health (WMH) Surveys With Sufficiently Large Samples for Country-Specific Analysis

eTable 3. Variation in Significant Multivariable Associations of Exposure to Civil Violence With Subsequent First Onset Anxiety, Mood, and Externalizing Disorder Onset as a Function of Number of Years Since First Exposure

eTable 4. Variation in Significant Multivariable Associations of Exposure to Civil Violence and Related Stressors With Relative Risk of Subsequent Anxiety, Mood, and Externalizing Disorder Onset as a Function of Whether the Respondent Was Still Living in the Country and Hostilities Were Ongoing or Ended or Whether the Respondent Immigrated to Another Country

eTable 5. Twelve-Month Persistence of *DSM-IV*/CIDI Disorders Among Lifetime Cases With Outsets at Least 2 Years Before Age at Interview and Relative Risk of 12-Month Disorder Persistence Associated With Being a Civilian in a War Zone

eTable 6. Relative Risk of 12-Month *DSM-IV*/CIDI Anxiety, Mood, and Externalizing Disorder Persistence Among Lifetime Cases With Outsets at Least 2 Years Before Age at Interview Associated With Being a Civilian in a War Zone and Related Stressors

eTable 7. Variation in Associations of Exposure to Civil Violence With Persistence of Subsequent Anxiety and Mood Disorders as a Function of Whether the Respondent Was Still Living in the Country and Hostilities Were Ongoing or Ended or Whether the Respondent Immigrated to Another Country

eTable 8. Variation in Association of Exposure to Civil Violence With 12-Month Persistence of Subsequent Anxiety, Mood, and Externalizing Disorders as a Function of Number of Years Since First Exposure

eReferences

This supplementary material has been provided by the authors to give readers additional information about their work.

© 2023 Axinn WG et al. *JAMA Network Open*.

eAppendix. Periods of Civil Conflicts in the Participating Countries

Overview

The following review of civil conflicts draws heavily on a series of general resources as well as on country-specific resources cited at the end of each section. The general resources used to provide information in each section include the data indicators system of the World Bank,[1] the United Nations Development Report,[2] the Oxford Our World in Data website,[3] the CIA World Factbook[4] and the online country reports from Amnesty International[5] and Human Rights Watch.[6]

Argentina (1973-1983)

The Argentine Dirty War (1973-1983) was a period of intensified civil conflict in Argentina launched by a right-wing military regime against suspected leftist dissenters. It began in 1973 when the return of the badly ailing but immensely popular former president Juan Peron from an 18-year exile in Spain precipitated hostilities between right and left-wing political factions, including those within Peron's own syncretic Justicialist Party. Violence began as soon as Peron arrived in the country, when right-wing Peronists opened fire into a massive crowd of mostly left-wing Peronists gathered to greet him at Ezeiza International airport, Buenos Aires, killing at least 13 and injuring more than 300. The aging Peron was soon re-elected president but died of natural causes less than one year later. In 1974, Isabel Martinez de Peron, his vice president and wife, assumed the presidency. However, she was increasingly sidelined by hardline military officers determined to suppress a growing left-wing insurgency fueled by discontent over a faltering economy. In 1976, she was overthrown by the right-wing Argentine military. The military junta known as the "National Reorganization Process" banned political parties and curtailed human rights. The junta conducted a systematic extrajudicial campaign of detention, torture, execution, and murder in the form of "disappearances" against perceived leftist political adversaries. Murders and torture – including beatings, electroshock, mutilation, starvation, and sexual abuse –were perpetrated at more than 500 detention centers. At one site alone – the "Navy Petty-Officers School of Mechanics" in Buenos Aires – an estimated 5,000 victims were murdered, leaving only 150 traumatized survivors. The scope and scale of state violence extended well beyond serious political opposition, including left-leaning workers, professionals, and students. The junta also abducted and raised hundreds of its victims' infants in an attempt to eradicate the intergenerational spread of their parents' political values. Speaking to an American journalist, General Jorge Rafael Videla, the junta's first leader, summarized this form of political eradicationism: "a terrorist is not just someone with a gun or a bomb but also someone who spreads ideas that are contrary to western civilization." The Argentine junta finally collapsed in 1983 following its defeat in the Falklands Islands War, launched to rally popular support that had plummeted owing to economic decline and political repression. At the "Trial of the Juntas" the new Argentine government sentenced dozens of former junta leaders for crimes against humanity, including General Videla, who died in prison while serving a life sentence. Former junta members are still being prosecuted, as long suppressed evidence is continually revealed. An estimated 30,000 Argentine civilians died in political violence during this period, with tens of thousands more tortured. While only 9,000 deaths were proven, Argentine government reports suggest a higher figure is probable, as tens of thousands of victims were "disappeared," murdered and disposed of with no trial or record.[7-11]

Colombia (1948-1958; 1964-present)

The Colombian Civil War "La Violencia" (1948-1958) was a series of widespread internal civil conflicts fought between the right wing Colombian Conservative Party, the Social Democratic Liberal Party, with more extreme conservative and Socialist paramilitary groups aligned with each. Following longstanding political tensions, civil war was triggered by the April 9, 1948, murder of Jorge Eliécer Gaitán, a charismatic left wing Colombian politician who was polling ahead in upcoming presidential elections against an incumbent Conservative president. Although the identity of Gaitán's murderer remains disputed, a Liberal Party run radio station immediately reported he had been assassinated by the Conservative Party government. This precipitated vast nationwide riots by Gaitan's supporters across Colombia, including the near total burning of the capital city of Bogota before order was restored by military intervention. This violence spiraled into an effective state of national civil war, with the Conservative government declaring a state of national emergency restricting freedoms. Although the Conservatives did not initially ban political opposition, the Liberal Party refused to participate in the 1950 presidential elections, which were heavily manipulated under martial law. The Conservatives then continued to engage in a combination of strikes, riots, and open civil insurrection. New Conservative Party president Laureano Gomez, elected under these dubious circumstances, then ruled as an effective dictator under state of emergency powers while attempting to suppress nationwide uprisings. Health problems forced his resignation in 1953. His successor was swiftly overthrown by a military coup led by General Gustavo Rojas Pinilla, another conservative seeking to entrench

© 2023 Axinn WG et al. *JAMA Network Open*.

conservative power while containing the spiraling civil chaos. Engaging in widespread human rights abuses, the military regime failed to prevent the ongoing civil war from worsening, with extremist right wing and communist factions further radicalizing the conflict. Finally, in 1957, a faction of moderate Conservative and Liberal politicians formed the "National Front," in which they agreed to share power, with Liberals and Conservative presidents elected in rotating four-year terms, after expelling the military regime. This system took effect in 1958, formally ending the Colombian Civil War. More than 200,000 Colombians – 3% of Colombia's pre civil war population – died in the ten-year conflict. At least two million Colombians were displaced from their homes as internal or external refugees.

The most recent Colombian Conflict (1964-Present) is a complex long term asymmetric civil conflict between factions of the Colombian government, criminal groups, and left and right-wing paramilitary organizations, often associated with or fronts for drug cartels and criminal gangs. The conflict also stems from demographic, socioeconomic, and ethnic divisions between urban and rural Colombians. The former, more likely to be either middle or upper class and of predominant European ancestry, have regularly supported conservative or moderate governments. The latter, typically poorer peasants of predominant Amerindian ancestry, have been the base of support for racial leftist rebel groups hoping to improve their status. However, violence has also occurred within communities that ostensibly share the same ethnic identity and politics. The conflict began when largely rural socialist and communist guerillas that had previously backed the Liberal Party in the Colombian Civil War continued fighting the National Front Government co-founded by their former allies. Many of these fighters remained deeply concerned about their basic rights as rural peasants in a highly classist society. Marxist-Leninist guerilla groups including the "National Liberation Army" (Spanish abbreviation ELN founded 1964), the Revolutionary Armed Forces of Colombia (FARC, founded 1966), and the Popular Liberation Army, (EPL, founded 1967) coalesced from guerilla and village self-defense groups that had fought in the Colombian Civil War. They continued to fight, now under a clearly Marxist, rather than broadly liberal ideological banner. The government dispatched army units to hunt and kill these guerillas. However, these units also regularly attacked and killed civilian villagers deemed sympathetic to the rebels. The government failed to fully defeat the rebels, who could easily escape and regroup in heavily jungled terrain with which they were familiar. The fighting continued for decades with no clear victory for either side in sight, despite the government holding the country's major population centers.

The rise of the international Drug Trade in the 1980s changed the nature of the civil conflict. Colombia is one of the few sources of South American coca leaves used to manufacture Cocaine, the most lucrative drug in the international drug trade. Criminal drug cartels rose to meet the new demand for cocaine, serving buyers in the United States. Rebel groups also began manufacturing and selling cocaine to fund their guerilla campaigns, but many also became involved in crime for profit, and the lines between political guerilla and simple criminals often blurred. During the 1980s, the United States under the Reagan Administration declared a global "War on Drugs," earmarking military aid to the drug war. Nonetheless, many drug cartels, including the largest, the Medellin Cartel, maintained links with the Colombian government. However, over time, extensive police efforts by the Colombian authorities, along with public fatigue with the violence, finally reduced the drug cartels' once near-monolithic power. The Medellin Cartel leader Pablo Escobar was killed by Colombian federal agents in 1993, and multiple guerilla groups and drug cartels were defeated or dispersed in the following decades. The World Bank reported that the Colombian intentional homicide rate dropped from a high of 84 per 100,000 people in 1991 to 23 per 100,000 people in 2020. Though this is still one of the highest murder rates in the world, it is now similar to the homicide rates in nearby countries like Brazil and Mexico and continues to decline.

The Colombian government, moderated and including many leftists, signed a peace agreement with FARC, the largest remaining rebel group, in 2016. In exchange for FARC laying down their arms, the government agreed to protect the rights of formal rebels and to provide funds to improve the social, economic, and health conditions of the rural communities in which FARC was based. A Truth Commission set up by the Colombian government in the wake of the 2016 peace agreement found that approximately 450,000 thousand people died in the Colombian conflict in the five previous decades. Fifty thousand people were kidnapped, typically by guerilla groups for ransom, between 1990 and 2018. Seven million Colombians were displaced from their homes. According to international monitoring agencies and human rights organizations, the Colombian conflict remains ongoing, including active major combat between government forces, drug cartels, and rebels, and continuing civilian casualties and dislocations.[12-15]

Lebanon: (1975-1990)

The Republic of Lebanon has been embroiled in civil conflict for much of its modern history. Although conflict occurred in the context of longstanding religious-sectarian division, the worse violence was linked with exogenous factors, primarily the Arab Israeli conflict and the Cold War. Lebanon is a heavily sectarian country, divided into more than a dozen major religious communities. The largest religious sects have historically been the Maronite Eastern Catholic Christians, Sunni and Shia Muslims, and Druze, along with smaller populations of

Alawites and multiple Christian sub-sects. These religious sects historically served as the primary units of social organization, community, and political collective action in what is now Lebanon. Following centuries of Ottoman rule, Lebanon's modern borders were drawn artificially in 1923, as a part of the larger French Mandate for Syria and Greater Lebanon.

The Republic of Lebanon was granted independence from the French Mandate in 1946. Post-independence Lebanon remained largely stable, despite Christian political over-representation and several instances of sectarian flare ups. Lebanon's later crises directly resulted from 100,000 Palestinian refugees fleeing the creation of the State of Israel. The Palestinians were neither granted Lebanese citizenship nor permitted by Israel to return to their homes. They were instead confined to refugee camps concentrated in Southern Lebanon, from which Palestinian militants launched recurrent attacks against Israel, often triggering cross-border Israeli retaliations. By the early 1970s, Lebanon housed approximately 300,000 stateless Palestinians, nearing 15% of its total population. Led by the Palestinian Liberation Organization (PLO), they seized effective control of much of Lebanon, establishing a *de facto* state in Palestinian refugee camps and a headquarters in Lebanon's capital, Beirut. The weak Lebanese military was largely powerless to intervene. The secular, left wing, Pan-Arabist PLO formed an alliance with endogenous left wing and Arabist Lebanese parties under the "Lebanese National Movement" (LNM). They were opposed by the "Lebanese Front" (LF) –an umbrella group of right-wing, overwhelmingly Maronite Christian militias fearing that an influx of non-Lebanese Muslims, Communists, and Arabists would destroy their community. In the Cold War context, the civil conflict developed between leftist secular militias backed by the Soviet Union and their Arab allies and right-wing Maronite militias backed by the West. However, the civil war's factionalism would become far more complex as the conflict progressed, particularly as foreign actors increasingly influenced events.

The Lebanese Civil War began officially on the morning of April 13, 1975, when an unknown gunman attacked a Maronite baptismal congregation in Beirut, killing four members of the LF and injuring one of its leaders, Pierre Gemayel. The LF retaliated later the same day by attacking a bus carrying both civilian and militant LNM affiliates, killing more than 20. Attack and counterattack followed, until full-scale civil war enveloped much of the country. Both the LF and the LNM murdered civilians in a series of targeted massacres in each of which hundreds of civilians perished. Among others, these included the LF perpetrated "Black Saturday" (Dec 5, 1975), Karantina (Jan 18, 76), and Tel al-Zaatar (Aug 76), and the LNM perpetrated Damour (Jan 20, 76), Chekka (July 5, 76), and Aishiyeh (Oct 21, 76). Intentional massacres of civilians and unarmed prisoners continued throughout the war. On August 16, 1982, in the largest single massacre, Maronite Lebanese Forces militias killed approximately 2,000 Palestinian civilians in Sabra and Shatila refugee camps in just one day. Lebanon as a whole was torn apart throughout the conflict. As the civil war began, Lebanon suffered near total socioeconomic collapse. Lebanon's GDP per capita in 1974 before the civil war was over $11,000, but by 1977, following two years of civil war, GDP had plummeted to only $3,000.

Lebanon's complex civil war lasted for 15 years, going through different stages and varying degrees of intensity, with heavy foreign involvement supporting much of the conflict. Syria (1976-2000) and Israel ("Peace for Galilee" invasion 1982-1985; occupation of southern Lebanon 1985-2005) invaded and occupied much of Lebanon both during and after the civil war. Israel retreated from Beirut in 1983, ended its main intervention in 1985, and abandoned a final small southern security buffer in 2005. The Lebanese civil war ended in October 1990 after Syrian, Druze, and Lebanese forces eliminated hardline Maronite resistors who rejected the signing of the Taif Peace Accords concluding the conflict.

Following the formal end of the civil war, Syria and a Syrian controlled puppet regime effectively occupied most of Lebanon until 2005. Syria then withdrew its forces and its proxy government collapsed following the "Cedar Revolution," of massive nationwide protests launched in response to the murder of the former Lebanese president, Rafik Hariri by Syrian agents. Lebanon remains highly politically volatile, with a weak central government and Hezbollah control over much of the country. The potential for future civil conflict is exacerbated by government corruption, gross economic mismanagement, and a massive refugee spillover from the ongoing Syrian civil war.[16-19]

Nigeria (1967-1970; 1997-present)

Nigeria experienced a major civil war and multiple major civil conflicts after gaining independence from the United Kingdom in 1963. Much of Nigeria's internal conflict owes to its post-colonial status. Its borders, artificially drawn under British colonialism, do not represent one historic nation with a shared language, culture, or identity. As a share of the population, Nigeria's four largest ethnolinguistic groups are the Hausa (30%), Yoruba (15.5%), Igbo (15.2%) and Fulani (6%).The Nigerian Civil War (1967-1970) began only several years after Nigeria attained full independence in 1963. The conflict was sparked in January 1966, when university educated Igbo military officers led a coup that killed democratically elected prime minister Abubakar Tafawa Balewa, claiming the former prime minister's government was grossly corrupt and incompetent, and that its officials were illegally

© 2023 Axinn WG et al. *JAMA Network Open*.

stealing Nigeria's oil wealth. Seeing that almost all the surviving coup leaders were Igbo and almost all their victims were non-Igbo, many Nigerians believed the coup was an Igbo ethnic plot to seize the country. As word of the coup spread, enraged non-Igbo northern Nigerians began targeting Igbo civilians in ethnic pogroms, killing tens of thousands, and causing more than a million to flee their homes. Northern Muslim soldiers mutinied against the new Igbo-led military regime only seven months after the coup. The Northerners overpowered and defeated the Igbo in a successful countercoup. Many of the counter-coup's leaders were initially planning on creating an independent Northern Nigerian state. However, civilian Nigerian officials convinced them it would be better for all Nigerians if Nigeria remained united. Most Igbo, feeling they would always be treated with hostility in a larger Nigeria, instead sought independence. The Igbo declared an independent State of Biafra in their historic coastal homeland on May 30, 1967. Nigeria did not accept this unilateral declaration, doubtlessly influenced by the fact that most of Nigeria's oil – its only major source of external revenue – was located in the territory of the self-declared Biafra. The Nigerian Civil War lasted from July 1967 and January 1970. The Biafrans were ultimately overwhelmed by Nigeria's much larger numbers, heavier military equipment, and foreign military support, and were forced to surrender. As many as 100,000 combatants died in the conflict, however the toll among Biafran civilians was far greater. A Nigerian naval blockade of Biafra resulted in as many as 2,000,000 Biafran civilian deaths, many of children, from famine, starvation, and disease. Many survivors, particularly those who were still developing children at the time, have suffered life-long health impairments from extended severe malnutrition. Approximately one in six Biafrans died in the three-year war. Total deaths in the conflict exceeded 5% of Nigeria's overall population the year the civil conflict began.

Although Nigeria has not subsequently faced another full-scale civil war on the scale of the Biafran conflict, Nigeria continues to encounter major ethnic and sectarian violence. These conflicts are exacerbated by public disaffection with political corruption and economic mismanagement, wealth inequality between the impoverished north and developing south, and difficulty sustaining a surging population, half of which is now under 18 years old. More than a million people have been displaced by violence in this region since conflicts began, out of a population of under 6 million.

Another, larger ongoing civil conflict, is the Boko Haram insurgency, an Islamist rebellion based in Northern Nigeria, extending to neighboring regions of Cameroon, Niger, Mali, and Chad. The rebellion began in 2009 when the hardline Islamist organization, "Boko Haram" – meaning "western education is banned" -- launched a violent uprising against the Federal Nigerian government in northern Nigeria. By 2014 the Nigerian military appeared unable to control the situation in the region. Boko Haram's links with international terrorist organizations, first Al Qaeda and later ISIL *(Islamic State in the Levant)*, caused the west to support the Nigerian government. A multinational western backed coalition of West African militaries formed against the group. The conflict remains active, with Boko Haram still launching effective raids across the north. Apart from Boko Haram's widespread campaign of terrorism, the Nigerian government has also been responsible for violence. Poorly trained and paid Nigerian soldiers and police have even functioned as bandits in regions of conflict, extorting money from civilians at gunpoint rather than fighting Boko Haram. The conflict has caused approximately 50,000 violent deaths, approximately 400,000 deaths from famine, and internally displaced 2.5 million people.[20,21]

Northern Ireland (1966-1998)

"The Troubles" was an extended low-level civil conflict fought in Northern Ireland between 1966 and 1998. Broadly, Irish Catholic Republican insurgents fought against the British government and Protestant militias in an attempt to unite Northern and Southern Ireland. Though the British military was tasked with preventing violence between both factions, it effectively sided with Protestants supporting continued British sovereignty over Northern Ireland. In addition to fighting in Northern Ireland, Irish Republican insurgents also perpetrated several terrorist attacks on the United Kingdom mainland and in Europe.

The Troubles developed beginning in the late 1960s. It resulted from growing Irish Catholic dissatisfaction with an overt system of legal discrimination imposed against them by the Protestant majority and hardline Protestant attempts to repress a growing Catholic civil rights movement. Northern Ireland had been under an effective state of martial law since its partition in 1922, with the "Civil Authorities Special Powers Act," originally designed to contain violence from militant Catholics opposed to partition at the time. This act granted the Protestant-controlled Home Affairs Office sweeping police powers, including the right to censor non-violent political expression, ban public gatherings, and indefinitely detain anyone to "maintain the order." Northern Irish Parliamentary districts were also overwhelmingly gerrymandered, disenfranchising Catholic voters. Although the Catholic share of Northern Ireland's population increased owing to their faster birthrate, their political representation did not. Catholic civil resistance emerged in the 1960s, calling to replaced gerrymandered districts with a one person one vote policy and end the martial law of the Civil Authorities Act. Fearing a revival of the defeated anti-Union Irish Republican Army, Pro-Union Protestants formed armed militias, beginning with the Ulster Protestant Volunteers (UVF) led by Ian

© 2023 Axinn WG et al. *JAMA Network Open*.

Paisley in 1966. These militias committed sporadic acts of violence and terrorism against Irish Catholic activists, beginning with several shootings and a bombing. They also violently disrupted peaceful Irish Catholic civil rights marches. Major rioting and violence erupted across Northern Ireland between August 12 and 16th, 1969, beginning when Protestant militias attacked Catholic protestors in the "Battle of Bogside," after which the Catholics defended themselves using rocks and Molotov cocktails. Mob violence then flared across the country, including multiple shootings. Eight people were killed, and hundreds were injured. The British military was deployed to maintain the peace. While this officially entailed dismantling both Catholic and Protestant paramilitaries, it focused almost exclusively on Catholics opposing UK membership. The Provisional Irish Republican Army, a radical splinter organization formed from the original IRA, emerged in December 1969. Many of its earliest members were called "69ers" because they were radicalized by the violence in 1969. Low level civil conflict continued for decades, as Catholic Irish nationalist insurgents sought Northern Ireland's adoption by the south. Combatants killed unarmed civilians throughout the conflict, primarily through bomb attacks.

The Troubles finally ended on December 2, 1999, with the signing of the Good Friday Agreement between the Provisional IRA, Ulster Protestant paramilitaries, the United Kingdom, and the Republic of Ireland. The agreement ended the anti-Catholic Civil Authorities Act and discriminatory policies that led to the conflict, without the UK ceding Northern Ireland. Approximately 3,500 people were killed and 50,000 injured over the entire 33-year conflict. Over half of the total fatalities – approximately 1,900 people – were civilians, with 1,049 British and Northern Irish soldiers and police, 162 Protestant paramilitaries, and 368 Irish Catholic paramilitaries also perishing. There was a spike of deaths in 1972, when 500 perished. Relative to population, fatalities from the Troubles approximated the intentional homicide rate in the United States during the 1990s crime wave.[22-24]

Peru (1980-2000)

Peru experienced a significant civil conflict concentrated in rural areas between 1980 and 2000, pitting rural far-left guerillas against the government and citizen militias, resulting in between 40,000 and 70,000 deaths before the insurgency was militarily quelled. Low-level conflict persisted in rural areas of the country. Very recently, more extensive civil unrest has resulted from the conservative congress' December 2022 removal of left-wing president Pedro Castillo, resulting in more than 50 civilian deaths and thousands of injuries as of January 2023, but these events occurred after the Peruvian WMH survey was completed.

From 1968 to 1980, Peru was controlled by a military junta called the "Revolutionary Government of the Armed Forces of Peru," which occurred in two stages. Initially led by General Juan Velasco Alvarado, the Peruvian military regime was a syncretic left-wing movement unlike any in Latin America at the time. The regime was oppressive and banned free speech but did not engage in human rights violations at the level of neighboring dictatorships. The regime's economic programs failed, causing a crippled economy, poverty, and violence. In 1975, the junta voted to replace an ailing Velasco with prime minister Brigadier General Francisco Morale Bermudez, who attempted to steer the Peruvian economy in a more capitalist direction in its second stage. When Bermudez's economic management also clearly failed, he and the rest of the junta agreed to cede power to a civilian government, allowing free elections in 1980. The 1980 elections were won by Fernando Belaunde, the center-left president who the military regime had overthrown in 1968.

Peru's civil conflict began when hardline leftist Marxist-Leninist-Maoist parties that had previously hoped to enact communism under the junta, refused to participate in the 1980 elections. These groups instead launched guerilla insurgencies in the impoverished rural areas of Peru where they were most popular, hoping to destabilize the Peruvian government and seize power. President Bermudez declared martial law and suspended constitutional rights in the rural Peruvian provinces where the rebels were strongest in 1981. However, military atrocities drew more supporters to its otherwise flagging cause and the insurgency continued to escalate. Alberto Fujimori won the 1990 presidential elections and immediately enacted sweeping economic reforms. Fujimori also established a large poverty relief fund, aware that his reforms would cause short-term economic shock. His neoliberal economic reforms allowed him to secure International Monetary Fund loans. The Peruvian economy began to recover. The major conflict was concluded by 2000 when President Fujimori was also forced to resign after investigations determined he had engaged in massive corruption. A Peruvian Truth and Reconciliation commission assembled between 2001 and 2003 concluded that approximately 70,000 Peruvians had died in insurgency related violence between 2000 and 2022.[25] Peru remains politically unstable and divided. Political protests are ongoing, with the congress' actions heavily condemned by leftwing neighboring Latin American states.[25,26]

South Africa (1948-1994)

The white minority-controlled government of the Republic of South Africa maintained "Apartheid," a formal legal system of race-based political repression and exclusion for nearly fifty years, before a peaceful transition to majority rule finally occurred 1994. "Apartheid," meaning "apartness" in the Afrikaans language spoken by a majority of South Africa's white population, conferred full political, social, and economic freedoms

© 2023 Axinn WG et al. *JAMA Network Open*.

only to individuals deemed racially "white," despite whites comprising less than 20% of South Africa's population during its imposition. Apartheid imposed an organized state of civil oppression restricting personal freedoms of non-whites, often through violent means.

Despite denying black South Africans *de jure* citizenship, the Apartheid government exploited black African labor. Low paid black African workers supported much of the South African economy while officially residing in Bantustans where no real jobs existed. The Apartheid government argued that black South Africans were actually enfranchised since they were technically "citizens" of these "states," however few were fooled. As de-colonization progressed across the rest of the world, South Africa became an international pariah state, condemned repeatedly at the UN and suffering severe economic sanctions. However, during the 1970s and 80s it retained significant western military support, as it was seen as a counterbalance to surrounding newly independent pro-Soviet African states with which it engaged in several long term "Border Wars." Apartheid South Africa also maintained universal adult military conscription for white males to maintain internal and external security.

Internal resistance to Apartheid initially occurred through peaceful protests and worker strikes. Violent resistance only began after the 1960 Sharpeville Massacre, in which white South African police wounded or killed hundreds of Black Africans protesting against restrictive pass laws. Another notable act of state violence was the Soweto uprising, in which hundreds of school children were shot protesting the introduction of Afrikaans in Black school curriculum mandated by the Apartheid state. Several Black resistance groups like the African National Congress (ANC) practiced both violent resistance, such as bombing Apartheid police stations, and peaceful protests. Apartheid internal security services clamped down on both armed insurgents and peaceful activists, with many instances of extrajudicial torture and killings. Many activists, like ANC leader Nelson Mandela who would later be South Africa's first Black President, received lengthy prison sentences for non-violent protests.

Apartheid finally collapsed as a result of both internal and external pressures. Global ostracization and sanctions heavily damaged the South African economy. Many of the best educated White South Africans emigrated from the country, causing "brain drain" further limiting the economy. Internal resistance rose from Black and increasingly large numbers of White South Africans. With the Cold War's conclusion, political isolation, growing economic crisis, and an end of western military support compelled the white-led South African government to make concessions in the early 1990s under the government of President F.W De Klerk, culminating in desegregated elections in 1994 leading to a victory by the ANC, the largest Black African opposition group during Apartheid.[27,28]

Other African Countries

A small number of WMH respondents (n=36), mostly interviewed either in South Africa or in one of the Western European WMH surveys, were exposed to sectarian violence in other African countries.

Algeria (1954-1962; 1991-2002): The Algerian War of Independence was one of the deadliest post-colonial revolutionary wars. Virtually all WMH respondents who were exposed to this war immigrated to France and participated in the French WMH survey. France originally occupied coastal Algeria, located across the Mediterranean Sea from Southern France, in 1830. By 1954, there were 1,400,000 French citizens living in Algeria, representing 13% of the total French population. French colonial rule in Algeria represented a particularly brutal form of ethno-religious Apartheid, as French-speaking Christians were privileged by the state in every possible way, able to vote, receiving full state benefits, and living and moving freely, whereas Arabic and Berber speaking Muslims were denied basic political, civil, and physical freedoms and were regularly subjected to atrocities. In 1945, after the end of World War II, French soldiers killed 6,000 unarmed Algerian civilians peacefully protesting for independence in the Setif and Guelma massacres. For the next decade, the French continued to hold onto Algeria, making few concessions. In 1962, after years of conflict, French President Charles De Gaulle granted Algeria full independence. Estimates of total deaths during the years of conflict vary wildly, ranging from an Algerian claim of 1.5 million to low-end French scholarly estimates of 400,000. However, as in most other similar colonial conflicts, civilian casualties from all causes – including famine, disease, and other violence surrounding civil breakdown – greatly exceeded military ones.

Angola, Guinea Bissau, and Mozambique (1961-1974): Revolutionary conflict occurred in Portuguese controlled Angola, Guinea Bissau, and Mozambique between 1961 and 1974, as communist African insurgents sought independence as People's Republics. Portugal's economy was also growing during the time of the colonial civil conflict, with local Africans also benefiting from higher living standards. The Portuguese successfully defeated the insurgencies in Angola and Mozambique, largely because local Africans supported them over the rebels, and actively assisted them. The situation in Guinea Bissau was different. The Guinea Bissauan revolutionaries, the "The African Party for the Independence of Guinea and Cape Verde" (Portuguese abbrev. PAIGC) were endogenous, popular, and effective combatants, well supported by Communist forces in newly independent pro-Soviet neighboring states. The Portuguese military never fully contained the Guinean uprising. The Portuguese Colonial

© 2023 Axinn WG et al. *JAMA Network Open*.

War ended in 1974, but the worst violence occurred following the Portuguese departure. In Guinea Bissau, PAIGC's hardline factions gained power over moderates. They massacred 7,500 pro-Portuguese African soldiers who had originally been promised amnesty under the peace treaty. In Angola and Mozambique, revolutionary movements backed by outside governments often functioning as little more than bandits, waged bloody warfare for decades. Virtually all the WMH respondents who were exposed to these conflicts immigrated to Portugal and were interviewed in the Portuguese WMH survey.

Congo (1960-1965): The Congo Crisis occurred immediately following the country's independence from Belgium in 1960. The conflict reflected common patterns in Cold War global politics and economic imperialism. Patrice Lumumba, a highly charismatic left-leaning pan-Africanist, was elected the Democratic Republic of Congo's first president in 1960. Almost immediately, African soldiers in the *Force Publique*, the former Belgian Congo's military force, revolted, expecting pay increases that did not materialize and feeling insulted by their white commander saying their role had not changed. The revolts spread throughout the force, accompanying mob violence against whites remaining in the Congo. There was widespread civil violence, escalating to a full civil war in the 1990s. Civil violence persists to this day, including ethnic clashes and the exploitation and abuse of Cobalt miners supplying most modern batteries. Virtually all WMH respondents who were exposed to this conflict immigrated to Belgium and were interviewed in the WMH Belgium survey.[29-32]

Other Latin American Civil Conflicts (1950-Present)

A small number of WMH respondents (n=49), mostly surveyed in the US or Mexico, were exposed to sectarian violence in Latin America. Multiple intense civil conflicts occurred throughout Latin America in the late twentieth century.

Mexico (1994-1996): The WMH survey used in this study was conducted in 2001, so does not account for and civil conflict after 2001. Between 1994 and 1996, Mexico experienced active civil conflict concentrated in the impoverished southernmost Mexican state of Chiapas. Chiapas' geographic isolation by tropical forests and mountain ranges allowed local people, primarily descended from the Mayans, to retain their languages and traditions. However, they remained persecuted and marginalized, beginning with forced labor under the Spanish Empire's encomienda system, extending to disenfranchisement, land theft, and state violence under the Republic of Mexico. During the 1990s, Mexico's autocratic long ruling Institutional Revolutionary Party implemented neoliberal economic reforms to improve a slowing economy. These included attempts to break up traditional communal farms in Chiapas, displacing historic Amerindian practices. The Chiapas rebellion began on January 1st, 1994, to coincide with the implementation of "NAFTA" the "North American Free Trade Agreement," opening Mexican markets to trade with the United States and Canada, that was opposed by Chiapas natives. Leftist rebels known as the "Zapatista Army of National Liberation," (EZLN, Spanish abbrev.) captured several communities. The rebellion was well publicized. The rebels received significant popular support from around the world. In no small part to avert negative press leading to reduced investment, the Mexican government agreed to meet some local demands for autonomy and native land rights in 1996.

Central America (1961-1996): Dictatorships emerged during the Cold War in El Salvador, Guatemala, Honduras, and Nicaragua supported by the West. Although in Nicaragua the communist Sandinista National Liberation Front overthrew the kleptocratic Somoza regime in 1979, clandestine military aid was provided for the next 11 years to right-wing militias and criminal gangs called the "Contras" fighting unsuccessfully to overthrow the communist regime. Right-wing military dictatorships in El Salvador and Guatemala used outside military aid both to fight violent leftist insurgencies and oppress regular citizens seeking democracy and social justice. In total 40,000 Nicaraguans, 80,000 Salvadorans, and as many as 200,000 Guatemalans are estimated to have perished in these conflicts. More than a million civilians were likely displaced due to violence. Outside military aid to right-wing dictatorships and movements ceased at the end of the Cold War, causing all to collapse within several years. A majority of Latin American nations have subsequently elected leftist governments, and several leftist Latin American guerilla groups still persist.

South America (1955-1991): In South America, anti-communist military dictatorships were installed and supported in Argentina, Bolivia, Brazil, Chile, Peru, and Uruguay during the Cold War, at the end of which they all quickly disintegrated. Although state violence officially targeted only armed leftist insurgents, these groups were small. *De facto*, most of this state terror was directed against non-violent leftist political activists, students, and trade unionists, as well as leftist political sympathizers without any real political involvements. South American military dictatorships are estimated to have killed at least 60,000 real or suspected leftists who were not fighting as armed insurgents at the time of their deaths. These regimes also detained more than 400,000 political prisoners, many innocent of any crimes, often for many years, who were often tortured during captivity.[33-35]

© 2023 Axinn WG et al. *JAMA Network Open*.

eTable 1. World Mental Health (WMH) Sample Characteristics by World Bank Income Categories[a]

Country by income category	Survey[b]	Sample characteristics[c]	Field dates	Age range	Part I[d]	Part II[d]	Response rate[e]
**I. Low and middle income countries**							
Brazil - São Paulo	São Paulo Megacity	São Paulo metropolitan area.	2005-8	18-93	5,037	2,942	81.3
Bulgaria	NSHS	Nationally representative.	2002-6	18-98	5,318	2,233	72.0
Bulgaria 2	NSHS - 2	Nationally representative.	2016-17	18-91	1,508	578	61.0
Colombia	NSMH	All urban areas of the country (approximately 73% of the total national population).	2003	18-65	4,426	2,381	87.7
Colombia – Medellin	MMHHS	Medellin metropolitan area	2011-12	19-65	3,261	1,673	97.2
Iraq	IMHS	Nationally representative.	2006-7	18-96	4,332	4,332	95.2
Lebanon	LEBANON	Nationally representative.	2002-3	18-94	2,857	1,031	70.0
Mexico	M-NCS	All urban areas of the country (approximately 75% of the total national population).	2001-2	18-65	5,782	2,362	76.6
Nigeria	NSMHW	21 of the 36 states in the country, representing 57% of the national population. The surveys were conducted in Yoruba, Igbo, Hausa and Efik languages.	2002-4	18-100	6,752	2,143	79.3
Peru	EMSMP	Five urban areas of the country (approximately 38% of the total national population).	2004-5	18-65	3,930	1,801	90.2
PRC[f] - Shenzhen[g]	Shenzhen	Shenzhen metropolitan area. Included temporary residents as well as household residents.	2005-7	18-88	7,132	2,475	80.0
Romania	RMHS	Nationally representative.	2005-6	18-96	2,357	2,357	70.9
South Africa[g]	SASH	Nationally representative.	2002-4	18-92	4,315	4,315	87.1
Ukraine	CMDPSD	Nationally representative.	2002	18-91	4,725	1,720	78.3
**TOTAL**					(61,732)	(32,343)	80.4
**II. High-income countries**							
Argentina	AMHES	Eight largest urban areas of the country (approximately 50% of the total national population).	2015	18-98	3,927	2,116	77.3
Australia[g]	NSMHWB	Nationally representative.	2007	18-85	8,463	8,463	60.0
Belgium	ESEMeD	Nationally representative. The sample was selected from a national register of Belgium residents.	2001-2	18-95	2,419	1,043	50.6
France	ESEMeD	Nationally representative. The sample was selected from a national list of households with listed telephone numbers.	2001-2	18-97	2,894	1,436	45.9

© 2023 Axinn WG et al. *JAMA Network Open.*

Country by income category	Survey[b]	Sample characteristics[c]	Field dates	Age range	Part I[d]	Part II[d]	Response rate[e]
**II. High-income countries**							
Germany	ESEMeD	Nationally representative.	2002-3	19-95	3,555	1,323	57.8
Israel	NHS	Nationally representative.	2003-4	21-98	4,859	4,859	72.6
Italy	ESEMeD	Nationally representative. The sample was selected from municipality resident registries.	2001-2	18-100	4,712	1,779	71.3
Japan	WMHJ 2002-2006	Eleven metropolitan areas.	2002-6	20-98	4,129	1,682	55.1
Netherlands	ESEMeD	Nationally representative. The sample was selected from municipal postal registries.	2002-3	18-95	2,372	1,094	56.4
New Zealand[g]	NZMHS	Nationally representative.	2004-5	18-98	12,790	7,312	73.3
N. Ireland	NISHS	Nationally representative.	2005-8	18-97	4,340	1,986	68.4
Poland	EZOP	Nationally representative	2010-11	18-65	10,081	4,000	50.4
Portugal	NMHS	Nationally representative.	2008-9	18-81	3,849	2,060	57.3
Qatar	WMHQ	Nationally representative. The sample was selected from a national list of cellular telephone numbers and restricted to Qatari nationals.[h]	2019-22	18-90	5,195	2,583	19.2[h]
Saudi Arabia[g]	SNMHS	Nationally representative	2013-2016	18-65	3,638	1,793	61.0
Spain	ESEMeD	Nationally representative.	2001-2	18-98	5,473	2,121	78.6
Spain-Murcia	PEGASUS-Murcia	Murcia region. Regionally representative.	2010-12	18-96	2,621	1,459	67.4
United States	NCS-R	Nationally representative.	2001-3	18-99	9,282	5,692	70.9
**TOTAL**					(94,599)	(52,801)	56.0
**III. TOTAL**					(156,331)	(85,144)	63.6

[a]The World Bank data were assessed at: http://data.worldbank.org/country at the time of each survey.

[b]NSHS (Bulgaria National Survey of Health and Stress); NSMH (The Colombian National Study of Mental Health); MMHHS (Medellín Mental Health Household Study); IMHS (Iraq Mental Health Survey); LEBANON (Lebanese Evaluation of the Burden of Ailments and Needs of the Nation); M-NCS (The Mexico National Comorbidity Survey); NSMHW (The Nigerian Survey of Mental Health and Wellbeing); EMSMP (La Encuesta Mundial de Salud Mental en el Peru); RMHS (Romania Mental Health Survey); SASH (South Africa Health Survey); CMDPSD (Comorbid Mental Disorders during Periods of Social Disruption); AMHES (Argentina Mental Health Epidemiologic Survey); NSMHWB (National Survey of Mental Health and Wellbeing); ESEMeD (The European Study Of The Epidemiology Of Mental Disorders); WMHJ2002-2006 (World Mental Health Japan Survey); NZMHS (New Zealand Mental Health Survey); NISHS (Northern Ireland Study of Health and Stress); EZOP (Epidemiology of Mental Disorders and Access to Care Survey); NMHS (Portugal National Mental Health Survey); WMHQ (World Mental Health Qatar Study);SNMHS (Saudi National Mental Health Survey); PEGASUS-Murcia (Psychiatric Enquiry to General Population in Southeast Spain-Murcia); NCS-R (The US National Comorbidity Survey Replication).

[c]Most WMH surveys are based on stratified multistage clustered area probability household samples in which samples of areas equivalent to counties or municipalities in the US were selected in the first stage followed by one or more subsequent stages of geographic sampling (e.g., towns within counties, blocks within towns, households within blocks) to arrive at a sample of households, in each of which a listing of household members was created and one or two people were selected from this listing to be interviewed. No substitution was allowed when the originally sampled household resident could not be interviewed. These household samples were selected from Census area data in all countries other than France (where telephone directories were used to select households) and the Netherlands (where postal registries were used to select households). Several WMH surveys (Belgium, Germany, Italy, Poland, Spain-Murcia) used municipal, country resident or universal health-care registries to select respondents without listing households. The Japanese sample is the only totally un-clustered sample, with households randomly selected in each of the 11 metropolitan areas and one random respondent selected in each sample household. 21 of the 32 surveys are based on nationally representative household samples.

[d]The WMH interviews in most countries were divided into two part to reduce respondent burden. Part I, which assessed core psychiatric disorders, was administered to 100% of respondents, whereas Part II was administered to 100% of Part I respondents who met criteria for any lifetime Part I disorder in addition to a probability subsample (typically 20-25%) of other Part I respondents. The records of noncertainty

© 2023 Axinn WG et al. *JAMA Network Open*.

Part I respondents were weighted by the inverse of their probability of selection into Part II to adjust for their under-sampling. This and other key WMH survey design features are discussed in detail elsewhere.[36]

[e]The response rate is calculated as the ratio of the number of households in which an interview was completed to the number of households originally sampled, excluding from the denominator households known not to be eligible either because of being vacant at the time of initial contact or because the residents were unable to speak the designated languages of the survey. The weighted average response rate is 63.6%.

[f]People’s Republic of China

[g]Although respondents earlier than age 18 were surveys, for purposes of cross-national comparisons, we limit the sample to those 18+ here.

[h]The survey began as a face-to-face household survey and had to switch to be phone-based due to the COVID-19 pandemic occurring shortly after the study started.

© 2023 Axinn WG et al. *JAMA Network Open*.

eTable 2. Significant Multivariable Associations of Exposure to Civil Violence and Related Stressors With Subsequent First Onset Anxiety, Mood, and Externalizing Disorders in World Mental Health (WMH) Surveys With Sufficiently Large Samples for Country-Specific Analysis[a]

	Any anxiety		Any mood		Any Externalizing		Number of respondents	
**Stressors**	**RR**	**(95% CI)**	**RR**	**(95% CI)**	**RR**	**(95% CI)**	**n_1**	**n_2**
I. Colombia								
Exposed to civil violence	2.1[b]	(1.5,3.0)	2.7[b]	(2.0,3.5)	2.7[b]	(1.9,3.8)	462	3433
Combatant	3.5[b]	(1.2,10.2)	1.0	(0.3,3.2)	2.5	(0.5,11.7)	21	0
Refugee	3.1[b]	(1.5,6.2)	0.8	(0.3,2.4)	1.3	(0.4,4.0)	24	0
χ^2_3		51.4[b]		49.2[b]		36.6[b]		
II. Lebanon								
Exposed to civil violence	3.6[b]	(1.9,6.9)	2.5[b]	(1.6,3.9)	0.9	(0.2,3.3)	516	265
Combatant	3.0[b]	(1.0,8.7)	1.0	(0.4,2.5)	2.0	(0.7,5.9)	34	0
Refugee	1.0	(0.5,1.9)	1.4	(0.9,2.1)	3.3[b]	(1.5,7.4)	272	0
χ^2_3		21.4[b]		29.5[b]		15.9[b]		
III. Northern Ireland								
Exposed to civil violence	1.8[b]	(1.3,2.4)	1.2	(0.9,1.6)	1.1	(0.8,1.6)	387	1344
Combatant	2.5[b]	(1.3,4.6)	0.6	(0.2,1.7)	1.0	(0.4,2.1)	23	0
Refugee	0.8	(0.4,1.7)	1.8	(0.8,4.4)	2.3[b]	(1.0,5.2)	15	0
χ^2_3		32.4[b]		5.9		5.7		
IV. South Africa								
Exposed to civil violence	1.6[b]	(1.1,2.3)	0.7	(0.4,1.3)	1.6[b]	(1.1,2.3)	286	3791
Combatant	2.9[b]	(1.1,7.4)	1.8	(0.4,9.3)	0.6	(0.2,1.8)	23	0
Refugee	0.1[b]	(0.0,0.6)	5.6[b]	(1.5,20.8)	1.7	(0.7,4.4)	29	0
χ^2_3		18.2[b]		11.6[b]		12.0[b]		

Abbreviations: RR, relative risk; 95% CI, 95% confidence interval of RR; n_1, the number of respondents who reported being exposed to civil violence; n_2, number of respondents who reported not being exposed to civil violence but who lived in the same country during the same time period (+/- 5 years of the time others in the same country were exposed) as those exposed.

[a]Based on the Multivariable discrete-time survival models in Table 4.

[b]Significant at the .05 level, two-sided test.

© 2023 Axinn WG et al. *JAMA Network Open*.

eTable 3. Variation in Significant Multivariable Associations of Exposure to Civil Violence With Subsequent First Onset Anxiety, Mood, and Externalizing Disorder Onset as a Function of Number of Years Since First Exposure[a]

	Any anxiety		Any mood		Any externalizing	
**Number of years[b]**	**RR**	**(95% CI)**	**RR**	**(95% CI)**	**RR**	**(95% CI)**
0-5	1.6	(0.7,3.6)	6.4[c]	(3.6,11.5)	3.7[c]	(1.3,10.5)
6-10	2.1[c]	(1.3,3.5)	3.1[c]	(2.1,4.8)	2.6[c]	(1.8,3.8)
11-15	2.6[c]	(1.8,3.6)	2.6[c]	(2.0,3.5)	3.0[c]	(2.2,3.9)
16-20	1.9[c]	(1.3,2.7)	1.9[c]	(1.4,2.6)	1.8[c]	(1.2,2.6)
21-25	1.9[c]	(1.2,3.0)	1.6[c]	(1.2,2.3)	2.0[c]	(1.3,3.0)
26-30	1.5[c]	(1.0,2.1)	1.0	(0.7,1.4)	1.1	(0.6,2.0)
31+	1.5[c]	(1.2,1.8)	0.9	(0.7,1.1)	0.9	(0.7,1.2)
χ^2_7	64.8[c]		124.0[c]		91.3[c]	
χ^2_6	9.8		79.1[c]		48.8[c]	

Abbreviations: RR, risk ratio; 95% CI, 95% confidence interval of RR.

[a]Based on the Univariable discrete-time survival models in Table 4 but with a decomposition of the dummy variable for exposure to civil violence by number of years since first exposure. These dummy variables were "turned on" at age of first occurrence and were time-variant across subsequent person-years.

[b]χ^2, tests of the significance of the associations between the stressor measures and the outcome. The χ^2_7 tests evaluated the significance of the set of 7 dummy variables for exposure by number of years between age of first exposure and age at interview, whereas the 6 degree of freedom χ^2_6 tests evaluated the significance of the differences across these 7 variables. The existence of significant variation in associations as a function of time since first exposure would be expected to result in a significant 6 degree of freedom test.

[c]Significant at the .05 level, two-sided test.

© 2023 Axinn WG et al. *JAMA Network Open*.

eTable 4. Variation in Significant Multivariable Associations of Exposure to Civil Violence and Related Stressors With Relative Risk of Subsequent Anxiety, Mood, and Externalizing Disorder Onset as a Function of Whether the Respondent Was Still Living in the Country and Hostilities Were Ongoing or Ended or Whether the Respondent Immigrated to Another Country[a]

	Any anxiety[c]		Any mood		Any externalizing	
**Subgroup[b]**	**RR**	**(95% CI)**	**RR**	**(95% CI)**	**RR**	**(95% CI)**
Exposed to organized civil violence						
Still living in country and …						
Hostilities ongoing	1.9[e]	(1.7,2.3)	1.6[e]	(1.4,1.9)	1.9[e]	(1.6,2.3)
Hostilities ended	1.2	(0.9,1.7)	1.1	(0.8,1.6)	0.6[e]	(0.3,1.0)
Immigrated to WMH country	0.9	(0.3,2.7)	1.0	(0.5,2.3)	0.3[e]	(0.1,0.9)
χ^2_3	68.0[e]		34.7[e]		57.8[e]	
χ^2_2	8.0[e]		4.7		26.6[e]	
Refugee[d]						
Still living in country and …						
Hostilities ongoing	NA	NA	1.4[e]	(1.0,1.9)	1.6[e]	(1.1,2.4)
Hostilities ended	NA	NA	2.5[e]	(1.3,4.5)	0.1	(0.0,1.1)
Immigrated to WMH country	NA	NA	2.1	(0.2,17.3)	0.0	--
χ^2_3	NA		12.5[e]		737.5[e]	
χ^2_2	NA		2.9		681.4[e]	

Abbreviations: RR, risk ratio; 95% CI, 95% confidence interval of RR.

[a]Based on the Multivariable discrete-time survival models in Table 4 but with a decomposition of the dummy variables for stressors into indicators of whether the hostilities were still ongoing or ended with the respondent still living in the country or whether the respondent emigrated from the country. These dummy variables were "turned on" at age of first occurrence of the stressor and were time-varying across subsequent person-years.

[b]Analyses were carried out in the entire sample, as in Table 5, but disaggregated each Table 5 predictor into 3 subgroups of person-years that differentiated respondents who were: (i) still living in their country of origin at the time of survey and hostilities were still ongoing in the country at that time (see eSupplement for dates); (ii) still living in their country of origin at the time of survey and hostilities had ended; and (iii) immigrated to a different country where they participated in the WMH survey. The χ^2_3 tests evaluated the significance of the set of 3 dummy variables, whereas the χ^2_2 tests evaluated the significance of the differences across these 3 variables. The existence of significant variation in associations as a function of whether hostilities were still ongoing, and the respondent emigrated from the country would be expected to result in a significant 2 degree of freedom test.

[c] Being a combatant was also significant in Table 4 for any anxiety disorder and was consequently included here as well. RR (95% CI) of being a combatant with anxiety disorder were 1.8 (0.2,3.5) for 0-12, 2.0[e] (1.3,3.2) for 13-21, and 2.7[e] (1.1,6.7) for 22+, with χ^2_3=13.3[e] and χ^2_3=0.4

[d]As shown in Table 4, being a refugee was unrelated to anxiety disorders and consequently was not included here in the model for anxiety disorders.

[e]Significant at the .05 level, two-sided test.

© 2023 Axinn WG et al. *JAMA Network Open*.

eTable 5. Twelve-Month Persistence of *DSM-IV*/CIDI Disorders Among Lifetime Cases With Outsets at Least 2 Years Before Age at Interview and Relative Risk of 12-Month Disorder Persistence Associated With Being a Civilian in a War Zone[a]

	Exposed[b]		Not Exposed[c]		Gross[d]		Net[e]		Sample Size[f]
Disorder	%	(SE)	%	(SE)	RR	(95% CI)	RR	(95% CI)	n
I. Anxiety disorder									
Generalized anxiety disorder	45.1	(4.3)	47.3	(2.0)	0.9	(0.6,1.1)	0.8	(0.6,1.1)	770
Panic and/or agoraphobia	57.7	(5.9)	55.5	(1.7)	1.0	(0.8,1.4)	1.0	(0.8,1.4)	938
Post-traumatic stress disorder	50.3	(4.0)	36.4	(2.3)	1.1	(0.8,1.4)	1.0	(0.8,1.4)	596
Specific phobia	80.4	(4.7)	73.8	(1.1)	1.0	(0.9,1.2)	1.0	(0.8,1.2)	1811
Social phobia	62.8	(5.9)	64.5	(1.7)	1.0	(0.8,1.4)	1.0	(0.8,1.4)	855
Any anxiety disorder	58.0	(2.2)	60.7	(0.7)	1.0	(0.9,1.1)	1.0	(0.9,1.1)	4970
II. Mood disorder									
Bipolar spectrum disorder	55.2	(6.5)	54.5	(2.7)	0.8	(0.6,1.1)	0.8	(0.6,1.1)	408
Major depressive disorder	37.9	(2.5)	42.1	(1.1)	0.9	(0.7,1.1)	0.9	(0.7,1.1)	2591
Any mood disorder	40.1	(2.3)	43.8	(1.0)	0.9	(0.7,1.1)	0.9	(0.7,1.0)	2999
III. Externalizing disorder									
Alcohol use disorder	21.6	(3.0)	27.3	(1.2)	0.8	(0.5,1.2)	0.8	(0.5,1.1)	1532
Illicit substance use disorder	25.5	(5.4)	22.8	(2.2)	1.2	(0.7,2.1)	1.1	(0.6,1.9)	447
Intermittent explosive disorder	56.4	(5.9)	58.1	(2.5)	0.9	(0.7,1.3)	0.9	(0.7,1.3)	452
Any externalizing disorder	28.3	(2.5)	31.5	(1.0)	0.9	(0.7,1.1)	0.9	(0.7,1.1)	2431
IV. Any disorder	42.9	(1.4)	47.9	(0.5)	1.0	(0.9,1.1)	0.9	(0.9,1.0)	10400

Abbreviations: RR, relative risk of 12-month disorder prevalence among respondents who were versus were not exposed; 95% CI, 85% confidence interval of RR.

[a]Based on stacked (across disorders) univariable person-level log link regression models controlling for country, respondent sex, age-of-onset of the focal disorder, and number of years since onset of the focal disorder.

[b]12-month prevalence of the disorder among respondents with a lifetime history of exposure to civil conflict as of age of first onset of the disorder.

[c]12-month prevalence of the disorder among respondents without a lifetime history of exposure to civil conflict as of age of first onset of the disorder.

[d]Gross = Controlling for age-of-onset of the focal disorder, number of years since onset of the focal disorder, country, and respondent sex.

[e]Net = Controlling for all variables in the gross models in addition to lifetime mental disorders as of the year before age-of-onset of the focal disorder.

[f](n) = Number of people with a lifetime history of the disorder two or more years before the survey.

© 2023 Axinn WG et al. *JAMA Network Open*.

eTable 6. Relative Risk of 12-Month *DSM-IV*/CIDI Anxiety, Mood, and Externalizing Disorder Persistence Among Lifetime Cases With Outsets at Least 2 Years Before Age at Interview Associated With Being a Civilian in a War Zone and Related Stressors[a]

Stressor	Any anxiety RR	Any anxiety (95% CI)	Any mood RR	Any mood (95% CI)	Any Externalizing RR	Any Externalizing (95% CI)
I. Univariable associations[b]						
Exposed to civil violence	1.0	(0.9,1.1)	0.9	(0.7,1.0)	0.9	(0.7,1.1)
Related stressors among civilians exposed to civil violence						
Refugee	1.1	(0.9,1.4)	1.1	(0.8,1.5)	0.6[d]	(0.3,1.0)
Saw atrocities	1.1	(0.8,1.4)	0.7	(0.4,1.1)	0.9	(0.5,1.5)
Combatant	0.7	(0.4,1.2)	0.9	(0.4,1.9)	0.5	(0.2,1.3)
Any of the above 3	1.0	(0.8,1.2)	1.0	(0.8,1.3)	0.7	(0.5,1.1)
II. Multivariable associations[c]						
Exposed to civil violence	1.0	(0.9,1.1)	0.9	(0.7,1.0)	0.9	(0.7,1.2)
Related stressors among civilians exposed to civil violence						
Refugee	1.1	(0.9,1.4)	1.3	(0.9,1.7)	0.6	(0.3,1.1)
Saw atrocities	1.1	(0.8,1.5)	0.7	(0.4,1.2)	1.1	(0.7,1.9)
Combatant	0.6	(0.4,1.2)	1.0	(0.5,2.2)	0.6	(0.2,1.4)

Abbreviations: RR, relative risk of 12-month disorder prevalence among respondents who were versus were not exposed; 95% CI, 95% confidence interval of RR.

[a]Based on stacked (across disorders) univariable person-level log link regression model controlling for country, respondent sex, age-of-onset of the focal disorder, and number of years since onset of the focal disorder.

[b]Only one of the four stressors (i.e., either exposure to civil violence, becoming a refugee, seeing atrocities, or becoming a combatant) included in the model.

[c]All stressors included in the model.

[d]Significant at the .05 level, two-sided test.

© 2023 Axinn WG et al. *JAMA Network Open*.

eTable 7. Variation in Associations of Exposure to Civil Violence With Persistence of Subsequent Anxiety and Mood Disorders as a Function of Whether the Respondent Was Still Living in the Country and Hostilities Were Ongoing or Ended or Whether the Respondent Immigrated to Another Country[a]

	Any anxiety		Any mood	
**Subgroup**	**RR**	**(95% CI)**	**RR**	**(95% CI)**
Still living in country and …[b]				
Hostilities ongoing	1.0	(0.7,1.5)	0.6	(0.3,1.1)
Hostilities ended	0.5	(0.1,1.7)	0.8	(0.4,1.9)
Immigrated to WMH country	1.0	(0.9,1.2)	0.9	(0.8,1.1)
χ^2_3		1.5		3.8
χ^2_2		1.3		1.9

Abbreviations: RR, relative risk of 12-month disorder prevalence among respondents who were versus were not exposed; 95% CI, 85% confidence interval of RR.

[a]Based on stacked (across disorders) univariable person-level log link regression model controlling for country, respondent sex, age-of-onset of the focal disorder, and number of years since onset of the focal disorder.

[b]χ^2, tests of the significance of the associations between the predictors and the outcome. The 3 degree of freedom χ^2_3 tests evaluated the significance of the set of 3 dummy variables for still living in the initial county with and without ongoing hostilities and emigration from the country, whereas the 2 degree of freedom χ^2_2 tests evaluated the significance of the differences across these 3 predictors.

© 2023 Axinn WG et al. *JAMA Network Open*.

eTable 8. Variation in Association of Exposure to Civil Violence With 12-Month Persistence of Subsequent Anxiety, Mood, and Externalizing Disorders as a Function of Number of Years Since First Exposure

	Any anxiety		Any mood		Any externalizing	
**Subgroup**	**RR**	**(95% CI)**	**RR**	**(95% CI)**	**RR**	**(95% CI)**
Age of first exposure[a]						
0-12	1.2	(1.0,1.4)	1.1	(0.8,1.4)	0.8	(0.5,1.2)
13-21	1.0	(0.8,1.3)	0.7[c]	(0.5,1.0)	0.8	(0.5,1.2)
22+	1.2	(0.9,1.5)	0.9	(0.7,1.2)	0.5	(0.2,1.7)
χ^2_3	5.0		5.3		2.0	
χ^2_2	1.7		4.4		0.6	
Number of years since first exposure[b]						
0-5	2.1[c]	(1.5,3.0)	1.6	(0.8,3.1)	0.2	(0.0,2.6)
6-10	0.8	(0.4,1.3)	0.8	(0.4,1.5)	1.1	(0.6,1.8)
11-15	1.2	(0.9,1.6)	0.7	(0.5,1.0)	0.8	(0.5,1.2)
16-20	1.0	(0.8,1.2)	0.8	(0.5,1.2)	0.6	(0.3,1.2)
21-25	1.1	(0.8,1.4)	0.8	(0.6,1.3)	0.6	(0.3,1.1)
26-30	1.1	(0.9,1.4)	0.8	(0.5,1.2)	1.4	(0.6,3.3)
31+	0.9	(0.8,1.1)	0.9	(0.7,1.1)	0.7	(0.4,1.3)
χ^2_7	20.8[c]		7.1		14.2[c]	
χ^2_6	18.4[c]		4.5		8.8	

Abbreviations: RR, risk ratio; 95% CI, 95% confidence interval of RR.

[a]χ^2, tests of the significance of the associations between the predictors and the outcome. The 3 degree of freedom χ^2_3 tests evaluated the significance of the set of 3 dummy variables for age-at-exposure compared to respondents who were never exposed to civil violence, whereas the 2 degree of freedom χ^2_2 tests evaluated the significance of the differences in the associations by age of first exposure within the subsample of respondents who were exposed to civil violence.

[b]χ^2, tests of the significance of the associations between the predictors and the outcome. The 7 degree of freedom χ^2_7 tests evaluated the significance of the set of 7 dummy variables for exposure by number of years between age of first exposure and age at interview, whereas the 6 degree of freedom χ^2_6 tests evaluated the significance of the differences across these 7 variables. The existence of significant variation in associations as a function of time since first exposure would be expected to result in a significant 6 degree of freedom test.

[c]Significant at the .05 level, two-sided test

© 2023 Axinn WG et al. *JAMA Network Open*.

eReferences

1. Indicators. World Bank. Accessed January 5, 2023. https://data.worldbank.org/indicator
2. Human Development Report 2021-22: Uncertain Times, Unsettled Lives: Shaping our Future in a Transforming World. UNDP (United Nations Development Programme). Accessed February 1, 2023. https://hdr.undp.org/content/human-development-report-2021-22
3. Ritchie H, Spooner F, Roser M. Our World in Data. Our World in Data. Accessed January 10, 2023. https://ourworldindata.org/
4. The World Factbook. Central Intelligence Agency. Accessed January 18, 2023. https://www.cia.gov/the-world-factbook/countries/
5. Countries. Amnesty International. Accessed January 3, 2023. https://www.amnesty.org/en/countries
6. Countries. Human Rights Watch. Accessed January 4, 2023. https://www.hrw.org/countries
7. Feierstein D. Political violence in Argentina and its genocidal characteristics. *Journal of Genocide Research*. 2006/06/01 2006;8(2):149-168. doi:10.1080/14623520600703024
8. *Centros clandestinos de la ciudad de Buenos Aires*. Instituto Espacio para la Memoria; 2007.
9. Argentina and Genocide Prevention Today. Auschwitz Institute for the Prevention of Genocide and Mass Atrocities. Accessed February 1, 2023. http://www.auschwitzinstitute.org/news/argentina-genprev-conference/
10. Truth Commission: Argentina. United States Institute of Peace. Accessed January 10 2023. https://www.usip.org/publications/1983/12/truth-commission-argentina
11. Juicio a Las Juntas Militares. International Crimes Database Accessed January 10, 2023. https://www.internationalcrimesdatabase.org/Case/1118/Juicio-a-las-Juntas-Militares/
12. Foundation WP. Colombia: La Violencia. Mass Atrocity Endings. Accessed December 20, 2022. https://sites.tufts.edu/atrocityendings/2016/12/14/colombia-la-violencia-2/
13. Murder rate plummets amid 'gangster peace' in Medellin. France 24. Accessed January 18, 2023. https://www.france24.com/en/live-news/20221014-murder-rate-plummets-amid-gangster-peace-in-medellin
14. Associated Press. Colombia Truth Commission Gives Scathing Report on Civil War. Voice of America (VOA News). Accessed December 20, 2022. https://www.voanews.com/a/colombia-truth-commission-gives-scathing-report-on-civil-war-/6637556.html
15. World Report 2022: Rights Trends in Colombia. Human Rights Watch. Accessed January 10, 2023. https://www.hrw.org/world-report/2022/country-chapters/colombia
16. Maktabi R. The Lebanese Census of 1932 Revisited. Who Are the Lebanese? *British Journal of Middle Eastern Studies*. 1999;26(2):219-241.
17. The Lebanese Civil War, 1975–1990. In: Schulhofer-Wohl J, ed. *Quagmire in Civil War*. Cambridge University Press; 2020:54-91.
18. United Nations Relief and Works Agency for Palestine Refugees. United Nations Relief and Works Agency for Palestine Refugees (UNRWA). https://www.unrwa.org
19. Makdisi S, Sadaka R. *The Lebanese Civil War, 1975–90*. 2005:59-86. *Understanding Civil War: Evidence and Analysis*. Accessed 2023/02/17/. http://www.jstor.org/stable/resrep02484.7
20. Stapleton TJ. Nigerian Civil War (1967–1970). *The Encyclopedia of War*. 2011.
21. Chronology of Important Events in the Nigerian Civil War. *The International Politics of the Nigerian Civil War, 1967-1970*. Princeton University Press; 1977:xv-xx.
22. Melaugh M. HMSO: Civil Authorities (Special Powers) Act (Northern Ireland), 1922. CAIN. Accessed January 7, 2023. https://cain.ulster.ac.uk/hmso/spa1922.htm
23. The Troubles. Encyclopædia Britannica, Inc. Accessed February 17, 2023. https://www.britannica.com/event/The-Troubles-Northern-Ireland-history
24. Battle of Bogside Marked 50 Years On. BBC News. Accessed December 30, 2022. https://www.bbc.com/news/uk-northern-ireland-foyle-west-49232799
25. Truth and Reconciliation Report. Comisión De La Verdad Y Reconciliación, Government of Peru. Accessed November 30, 2022. http://www.cverdad.org.pe/ingles/pagina01.php
26. Rendon S. Capturing correctly: A reanalysis of the indirect capture–recapture methods in the Peruvian Truth and Reconciliation Commission. *Research & Politics*. 2019;6(1):2053168018820375. doi:10.1177/2053168018820375
27. Truth and Reconciliation Commission Republic of South Africa Department of Justice. https://www.justice.gov.za/trc

© 2023 Axinn WG et al. *JAMA Network Open*.

28. Welsh F. *A History of South Africa*. HarperCollins; 2000.
29. Stapleton TJ. *A military history of South Africa : from the Dutch-Khoi wars to the end of apartheid*. Praeger; 2010.
30. Adam H, Barbara K. *King Leopold's Ghost: A Story of Greed, Terror, and Heroism in Colonial Africa*. Mariner Books/Houghton Mifflin Harcourt; 2020.
31. Vanthemsche G. *Belgium and the Congo, 1885–1980*. Cambridge University Press; 2012.
32. Howe M. Portuguese Settlers in Africa Lead the Good Life. The New York Times. Accessed January 15, 2023. https://www.nytimes.com/1972/08/08/archives/portuguese-settlers-in-africa-lead-the-good-life.html
33. Human Rights Watch World Report 1992 - Mexico. Human Rights Watch. Accessed February 1, 2023. https://www.refworld.org/docid/467fca49c.html
34. Bevins V. *The Jakarta Method: Washington's Anticommunist Crusade and the Mass Murder Program that Shaped Our World*. Public Affairs; 2020.
35. Kornbluh P. *The Pinochet File: A Declassified Dossier on Atrocity and Accountability*. The New Press; 2013.
36. Kessler RC, Heeringa SG, Pennell BE, Mneimneh Z, S.A. C, Zaslavsky AM. Methods of the World Mental Health Surveys Department of Health Care Policy, Harvard Medical School. Accessed February 23, 2023. http://www.hcp.med.harvard.edu/wmh/ftpdir/WMHMethods.pdf

© 2023 Axinn WG et al. *JAMA Network Open*.
