# Supplementary material for: Findings From the World Mental Health Surveys of Civil Violence Exposure and Its Association With Subsequent Onset and Persistence of Mental Disorders
Source: JAMA Netw Open. 2023 Jun 20;6(6):e2318919. doi: 10.1001/jamanetworkopen.2023.18919 (PMC10282884; doi:10.1001/jamanetworkopen.2023.18919)
Supplement: Supplement 2. — Nonauthor Collaborators [file jamanetwopen-e2318919-s002.pdf]

Supplement 2. Nonauthor Collaborators
*First name, last name, and suffix (if applicable) are required and will appear in PubMed.

*Group Name(s): WHO World Mental Health Survey Collaborators							
***First Name and Middle Initial(s)**	***Last Name**	***Suffix (eg, Jr, III)**	Academic Degrees	Institution	Location (city, state/province, country)	Role or Contribution, eg, chair, principal investigator	Group (if more than 1 Group listed in the byline) and/or Subgroup (eg, Steering Committee)
Ali	Al-Hamzawi		MD	College of Medicine, Al-Qadisiyah University	Diwaniyah Governorate, Iraq		
Yasmin A.	Altwaijri		PhD	Epidemiology Section, King Faisal Specialist Hospital and Research Center	Riyadh, Saudi Arabia		
Laura Helena	Andrade		MD, PhD	Núcleo de Epidemiologia Psiquiátrica-LIM 23, Instituto de Psiquiatria Hospital das Clinicas da Faculdade de Medicina da Universidade de São Paulo	São Paulo, Brazil		
Lukoye	Atwoli		MD, PhD	1. Department of Mental Health and Behavioral Sciences, College of Health Sciences, School of Medicine, Moi University; 2. Brain and Mind Institute and Medical College East Africa, the Aga Khan University	1. Eldoret, Kenya; 2. Nairobi, Kenya		
Corina	Benjet		PhD	Department of Epidemiologic and Psychosocial Research, National Institute of Psychiatry Ramón de la Fuente Muñiz	Mexico City, Mexico		
Guilherme	Borges		PhD	National Institute of Psychiatry Ramón de la Fuente Muñiz	Mexico City, Mexico		
Evelyn J.	Bromet		PhD	Department of Psychiatry, Renaissance School of Medicine, Stony Brook University	Stony Brook, NY, USA		
Somnath	Chatterji		MD	Department of Information, Evidence and Research, World Health Organization	Geneva, Switzerland		
Louisa	Degenhardt		PhD	National Drug and Alcohol Research Centre, University of New South Wales	Sydney, NSW, Australia		
Giovanni	de Girolamo		MD	IRCCS Istituto Centro San Giovanni di Dio Fatebenefratelli	Brescia, Italy		

Supplement 2. Nonauthor Collaborators
*First name, last name, and suffix (if applicable) are required and will appear in PubMed.

***First Name and Middle Initial(s)**	***Last Name**	***Suffix (eg, Jr, III)**	Academic Degrees	Institution	Location (city, state/province, country)	Role or Contribution, eg, chair, principal investigator	Group (if more than 1 Group listed in the byline) and/or Subgroup (eg, Steering Committee)
Josep Maria	Haro		MD, PhD	1. Research, Teaching and Innovation Unit, Parc Sanitari Sant Joan de Déu, Sant Boi de Llobregat; 2. Centre for Biomedical Research on Mental Health (CIBERSAM); 3. Departament de Medicine, Universitat de Barcelona	1. Barcelona, Spain; 2. Madrid, Spain; 3. Barcelona, Spain		
Meredith G.	Harris		PhD	1. School of Public Health, The University of Queensland; 2. Queensland Centre for Mental Health Research, The Park Centre for Mental Health	1. Herston, QLD 4006, Australia; 2. QLD 4072, Australia		
Hristo	Hinkov		MD, PhD	National Center for Public Health and Analyses	Sofia, Bulgaria		
Chi-yi	Hu		MD, PhD	Shenzhen Institute of Mental Health and Shenzhen Kangning Hospital	Shenzen, China		
Peter	de Jonge		PhD	1. Department of Developmental Psychology, University of Groningen; 2. Interdisciplinary Center for Psychopathology and Emotion Regulation, University Medical Center Groningen	1. Groningen, The Netherlands; 2. Groningen, The Netherlands		
Aimee Nasser	Karam		PhD	Intitute for Development, Research, Advocacy and Applied Care (IDRAAC)	Beirut, Lebanon		
Georges	Karam		MD	1. Department of Psychiatry and Clinical Psychology, St George Hospital University Medical Center, University of Balamand, Faculty of Medicine; 2. Institute for Development, Research, Advocacy and Applied Care (IDRAAC)	1. Beirut, Lebanon; 2. Beirut, Lebanon		
Alan E.	Kazdin		PhD	Department of Psychology, Yale University	New Haven, CT, USA		
Norito	Kawakami		MD, DMSc	Department of Mental Health, Graduate School of Medicine, The University of Tokyo	Tokyo, Japan		
Salma	Khaled		PhD	Social and Economic Survey Research Institute, Qatar University	Doha, Qatar		
Andrzej	Kiejna		MD, PhD	Faculty of Applied Studies, University of Lower Silesia	Wroclaw, Poland		

Supplement 2. Nonauthor Collaborators

*First name, last name, and suffix (if applicable) are required and will appear in PubMed.

***First Name and Middle Initial(s)**	***Last Name**	***Suffix (eg, Jr, III)**	Academic Degrees	Institution	Location (city, state/province, country)	Role or Contribution, eg, chair, principal investigator	Group (if more than 1 Group listed in the byline) and/or Subgroup (eg, Steering Committee)
John J.	McGrath		MD, PhD	1. Queensland Centre for Mental Health Research, The Park Centre for Mental Health; 2. Queensland Brain Institute, The University of Queensland; 3. National Centre for Register-based Research, Aarhus University	1. Wacol, QLD 4072, Australia; 2. St Lucia, QLD 4065, Australia; 3. Aarhus V 8000 Denmark		
Maria Elena	Medina-Mora		PhD	National Institute of Psychiatry Ramón de la Fuente Muñiz	Mexico City, Mexico		
Jacek	Moskalewicz		PhD	Institute of Psychiatry and Neurology	Warsaw, Poland		
Fernando	Navarro-Mateu		MD, PhD	1. Unidad de Docencia, Investigación y Formación en Salud Mental (UDIF-SM), Gerencia Salud Mental, Servicio Murciano de Salud; 2. Murcia Biomedical Research Institute (IMIB-Arrixaca); 3. CIBER Epidemiology and Public Health-Murcia (CIBERESP-Murcia)	1. Murica, Spain; 2. Murcia, Spain; 3. Murica, Spain		
Daisuke	Nishi		MD, PhD	Department of Mental Health Graduate School of Medicine, The University of Tokyo	Tokyo, Japan		
Margreet	ten Have		PhD	Trimbos-Instituut, Netherlands Institute of Mental Health and Addiction	Utrecht, The Netherlands		
Maria Carmen	Viana		MD, PhD	Department of Social Medicine, Postgraduate Program in Public Health, Federal University of Espírito Santo	Vitoria, Brazil		
Daniel V.	Vigo		MD, DrPH	1. Department of Psychiatry, University of British Columbia; 2. Department of Global Health and Social Medicine, Harvard Medical School	1. Vancouver, BC, Canada; 2. Boston, MA, USA		
Cristian	Vladescu		MD, PhD	National Institute for Health Services Management	Bucharest, Romania		
Bogdan	Wojtyniak		ScD	Centre of Monitoring and Analyses of Population Health, National Institute of Public Health-National Research Institute	Warsaw, Poland		

Supplement 2. Nonauthor Collaborators
*First name, last name, and suffix (if applicable) are required and will appear in PubMed.

***First Name and Middle Initial(s)**	***Last Name**	***Suffix (eg, Jr, III)**	Academic Degrees	Institution	Location (city, state/province, country)	Role or Contribution, eg, chair, principal investigator	Group (if more than 1 Group listed in the byline) and/or Subgroup (eg, Steering Committee)
Peter	Woodruff		MBBS, PhD, FRCPsych	Department Neuroscience, University of Sheffield	Sheffield, UK		
Miguel	Xavier		MD, PhD	Lisbon Institute of Global Mental Health and Chronic Diseases Research Center (CEDOC), NOVA Medical School|Faculdade de Ciências Médicas, Universidade Nova de Lisboa	Lisbon, Portugal		
Alan M.	Zaslavsky		PhD	Department of Health Care Policy, Harvard Medical School	Boston, MA, USA		
