# Supplementary material for: Findings From the World Mental Health Surveys of Civil Violence Exposure and Its Association With Subsequent Onset and Persistence of Mental Disorders
Source: JAMA Netw Open. 2023 Jun 20;6(6):e2318919. doi: 10.1001/jamanetworkopen.2023.18919 (PMC10282884; doi:10.1001/jamanetworkopen.2023.18919)
Supplement: Supplement 3. — Data Sharing Statement [file jamanetwopen-e2318919-s003.pdf]

Data Sharing Statement

Axinn. Findings From the World Mental Health Surveys of Civil Violence Exposure and Its Association With Subsequent Onset and Persistence of Mental Disorders. *JAMA Netw Open*. Published June 20, 2023. doi:10.1001/jamanetworkopen.2023.18919

Data
Data available: Yes

Which data are available: Access to the cross-national World Mental Health (WMH) data is governed by the organizations funding and responsible for survey data collection in each country. These organizations made data available to the WMH consortium through restricted data sharing agreements that do not allow us to release the data to third parties. The exception is that the U.S. data are available for secondary analysis via the Inter-University Consortium for Political and Social Research (ICPSR), https://www.icpsr.umich.edu/icpsrweb/ICPSR/series/00527.
